# Opportunities for Expanding Access to Veterinary Care: Lessons From COVID-19

**DOI:** 10.3389/fvets.2022.804794

**Published:** 2022-04-11

**Authors:** Sage M. Smith, Zachary George, Colleen G. Duncan, Danielle M. Frey

**Affiliations:** ^1^College of Veterinary Medicine and Biomedical Sciences, Colorado State University, Fort Collins, CO, United States; ^2^College of Veterinary Medicine and Biomedical Sciences, Department of Microbiology, Immunology and Pathology, Colorado State University, Fort Collins, CO, United States; ^3^College of Veterinary Medicine and Biomedical Sciences, Office of the Dean, Colorado State University, Fort Collins, CO, United States

**Keywords:** access to veterinary care, pandemic (COVID19), vulnerable population, telemedicine, veterinary

## Abstract

The COVID-19 pandemic impacted people and professions around the world, including veterinary medicine. The epidemiology of SARS-CoV-2 broadened the definition of vulnerability in human populations, and the virus' economic impacts exacerbated well-established financial barriers to providing equal access to medical care. The objective of this study was to explore how the pandemic was impacting access to companion animal care in the months March-September of 2020, with a focus on traditionally vulnerable as well as newly vulnerable populations. Additionally, this study sought to identify areas on which the veterinary profession can focus in order to help increase access to veterinary care, including the veterinary school curriculum, continuing education, and telemedicine. We conducted surveys and interviews with animal owners (*n* = 1009), veterinarians and clinic staff (*n* = 516), and access to veterinary care organizations (*n* = 17). Collectively, these responses highlighted how the COVID-19 pandemic created new, and amplified existing, issues with accessing and providing veterinary care. Three critical themes arose; (1) opportunities for further learning for the veterinary profession; including curricula around telemedicine, financially resilient business models and understanding health disparities and vulnerable populations; (2) a need for a network of collaboration and communication across veterinary clinics and access to care organizations and (3) future preparedness for health, economic or other crises response. Overall, the pandemic emphasized the complexity of access to care, as well as the role of veterinarians in public health. This information can be used to develop strategies to aid in increased access to veterinary care now and in the face of future disasters.

## Introduction

When attempting to access veterinary care, some pet owners experience barriers which prohibit them from supporting the health of their pet. The most common barriers include cost, accessibility of care (including location and transport), veterinarian-client communication, culture or language, and lack of client education (1, 2). The inability of pet owners to receive veterinary care for their animals can have a direct negative impact on animal welfare (3). Lack of veterinary care also presents a public health threat, as poor animal health can directly affect human health by increasing the risk of zoonotic and vector-borne diseases (4, 5). Finally, pet ownership has a significant and positive impact on mental health, and threats to the health of pets can impact pet owners negatively (5, 6). The barriers to accessible veterinary care and the complexity of their impact and origin have been well-described by Lem et al. through a framework that shows the interrelated nature of human, animal, environmental and socioeconomic factors (2). Work has also been done to begin the process of associating the social determinants of human health to social determinants of animal health (5). Despite this established framework and other work in the field, barriers persist and there is a continued need for further empirical research on how to provide accessible veterinary health care (1).

As with all health and economic disparities, certain populations of vulnerable people experience the burden of these barriers to veterinary care more than others. Vulnerable human populations have traditionally been considered as groups and communities that experience barriers to economic, political, social, and environmental resources, leaving them at higher risk for health issues (7). These same barriers to resources may place their pets at higher risk for health inequalities as well (5). The lack of resources that these populations experience results in decreased resilience and increased adversity in the face of extreme events (8). Vulnerable populations suffer greater consequences during economic downturns as well as health crises (9, 10). Abandonment of pets is common when owners are faced with socio-economic challenges, (11) eviction and disaster situations (12). Additionally, economic recessions have been shown to have direct implications for companion animals (13, 14). These findings suggest that companion animals in vulnerable populations are at higher risk of negative outcomes during disaster situations and extreme events.

On March 11, 2020, the World Health Organization declared Coronavirus disease 19 (COVID-19) caused by the virus, severe acute respiratory syndrome coronavirus 2 (SARS-CoV-2) a global pandemic (15). During its course, this health and economic crisis has exacerbated the underlying health, economic, and social disparities that vulnerable populations already faced. Widespread reporting showed early on that ethnic minorities, Native Americans, and low-income communities have been disproportionately affected by the virus compared to the United States' population at large (16, 17). COVID-19 has also triggered the emergence of groups of “newly” vulnerable people who were not considered so at the onset of the pandemic. These newly vulnerable groups are now struggling in their abilities to shoulder the financial and physical burdens brought about by this crisis, thus reshaping how vulnerability is being defined (18). Some of the individuals who are now defined by their “newly” vulnerable status are people who are considered high risk for severe COVID-19 illness, including adults over 65 and people with certain pre-existing health conditions (19). These as well as individuals who lack health insurance and/or employment, may or may not have been deterred by the common hurdles to accessing veterinary care prior to the pandemic. However, in the face of COVID-19, these newly vulnerable groups may now additionally face the unique barriers of the personal health risk and financial hardships of going into a veterinary clinic. We hypothesized that the pandemic caused additional, and some unexpected, stressors for those in need of, and providing, veterinary care. The objective of this study was to explore how the pandemic has impacted access to companion animal care in the United States of America, with a focus on traditionally vulnerable as well as newly vulnerable populations. We assessed this through inquiries with three stakeholders, pet owners, veterinarians and clinic staff and access to care organizations. Additionally, this study aimed to identify areas on which the veterinary profession can focus in order to help increase access to veterinary care, including the veterinary school curriculum, continuing education, and telemedicine. This information can be used to inform strategies to aid in the increased access to veterinary care.

## Materials and Methods

To better understand the impact of the pandemic on pet owners, including vulnerable populations, a series of surveys and key informant interviews were conducted. All surveys and interview scripts were reviewed by Colorado State University's Institutional Review Board and determined to be exempt, meaning that the studies posed no more than minimal risk to human participants and fell into a category of exempt research. All studies began with obtaining either written or verbal consent, and all research data collected was published anonymously. Descriptive and comparative statistics were conducted using commercially available software. As most survey questions did not require a response, individual unanswered questions were excluded from analysis.

### Pet Owners

An anonymous online survey (Supplementary Material 1) was developed using Qualtrics software and disseminated through Amazon's Mechanical Turk (MTurk). MTurk is an online platform designed for people (“workers”) to sign up to complete virtual tasks for compensation. Participants were compensated one dollar for completion of the survey. Inclusion criteria were that the respondents were 18 years or older, the primary caretaker of a dog and/or cat that needed veterinary care during the pandemic, and that in the past 3 years prior to the COVID-19 pandemic, they had taken this dog and/or cat to the veterinarian. Income groups were divided based on methods used in the 2018 Access to Veterinary Care report published by the University of Tennessee (20) which used guidelines that determine eligibility for certain federal aid programs. Household income categories included those below 138% of federal poverty level (criteria for Medicaid and the Supplemental Nutritional Assistance Program qualification), those between 138 and 250% of federal poverty level (criteria for Cost Sharing Reduction Subsidies qualification), and those above 250% of federal poverty level. The 2020 federal poverty level guidelines were used, and incomes were rounded to the nearest $500 for simplicity. The definition for people at high risk for severe COVID-19 illness was taken from the Center for Disease Control and Prevention (CDC) website on June 4, 2020 (19).

The survey had 22 questions spanning 4 broad categories: demographics, impact of the pandemic on the ability to care for pets, owners' perceptions, and telemedicine. Responses were required for consent and inclusion criteria only. Certain data was analyzed by categorizing vulnerable vs. non-vulnerable pet owners and looking at differences in answers. People were considered vulnerable if they met ANY of the following criteria: self-identified as high risk for severe COVID-19 illness based on CDC definition as of June 4, 2020, household income fell below 250% of the federal poverty level, race/ethnicity was reported as either Black/African American, Hispanic/Latino, or Native American (21), employment was lost during the pandemic, respondent did not have health insurance, or only had it during part of the study period, and respondents who reported that inability to use public transportation or inability to access a car made it more challenging to go to the veterinarian during the pandemic. The survey was dispersed on MTurk in 3 batches, on July 10, 2020, July 16, 2020, and July 17, 2020. If multiple responses were recorded by a single MTurk user, only the first response was included in analysis. When questions allowed for an “other” answer option to be written in, answers were recategorized into existing options or reported separately if 10% or greater of write-ins reported a similar answer.

### Veterinarians

An anonymous online survey (Supplementary Material 2) was developed for small animal veterinarians, technicians, and office managers and shared through opportunistic dissemination of the survey link through veterinary associations (ex. Veterinary Medical Associations, specialty colleges and social media groups targeting animal health professionals). The survey consisted of 25 questions divided into 4 categories: demographics, access to veterinary care for traditionally vulnerable populations, access to veterinary care for clients at high risk for COVID-19, and telemedicine. Participation in the survey was incentivized by offering the opportunity to win one of ten $50 gift cards available to the first 200 respondents. The survey was accessible from June 24-September 24, 2020. When questions allowed for an “other” answer option to be written in, answers were recategorized into existing options or reported separately if more than 10% of write-ins reported a similar answer. Fully open-ended questions were analyzed for similar answers/themes, and if 10% or greater of the answers fell into a similar theme, the answer was reported. Survey participants were provided with the following definition for “traditionally” vulnerable populations, “Vulnerable human populations have traditionally been considered as groups and communities that experience barriers to economic, political, social, and environmental resources, leaving them at higher risk for health issues for themselves and their pets. These include, but are not limited to, people experiencing homelessness, the elderly, and low-income communities.” Additionally the survey outlined the definition for “newly” vulnerable populations; “The CDC defined higher risk populations for severe illness from COVID-19 as people 65 years and older, people who live in a nursing home or long-term care facility, people with chronic lung disease or moderate to severe asthma, people with serious heart conditions, people who are immunocompromised, severely obese people, people with diabetes, people with chronic kidney disease undergoing dialysis, and people with liver disease” (Supplementary Material 2).

### Access to Care Organizations

A series of key informant interviews were used to explore the challenges the organizations and the vulnerable communities they serve were facing and provided an opportunity to potentially capture responses not available through the use of online survey tools (see limitations). Professionals involved with organizations that focus on providing access to veterinary care (i.e., Spay/neuter, preventive, or emergency services) or animal-assisted therapy to vulnerable populations and communities were contacted. Contacts were made through a network of professional connections or by reaching out through contacts listed on websites, with emphasis on a variety of types of vulnerable populations being represented. Twenty-six organizations were contacted, and 17 professionals, representing 20 groups, were ultimately interviewed about the impact of the COVID-19 pandemic on their ability to provide companion animal care to the communities they support. Several professionals were a part of more than one organization and therefore the number of organizations represented (20) was larger than the number of interviewees. Two organizations were represented by two different interviewees. The interview questions were divided into four broad categories: barriers to care and pandemic services; resources and support; challenges experienced and concerns; and successes and opportunities for future. The first question of the interview was meant for introduction and context; therefore, responses were not included in the analysis. There were 18 open-ended questions overall (Supplementary Material 3). Interviews were conducted by a single author over video or telephone calls typically lasting from 30 min to 1 h. Interviews were conducted from July 24 to September 27, 2020. Answers to the interview questions were transcribed by the interviewing author. Two authors then independently analyzed the responses for common, broad topics and met to reach consensus on emergent themes.

## Results

### Pet Owner Survey

#### Demographics

After eliminating repeat responses and those that were <25% completed, a total of 1,009 responses were included in the final analysis. Respondents were asked to identify the state they live in, and then the regional definitions put forward by the U.S. Census Bureau were used to group respondents into four regions: Northeast, South, Midwest, and West. The South had the most respondents with 40% (406/1009), followed by the West (22%, 221/1009), the Midwest (19%, 192/1009), and the Northeast (19%, 190/1009). The majority of respondents were White (78%, 785/1009), followed by Black or African American (8%, 83/1009), Asian or Pacific Islander (7%, 70/1009), Hispanic or Latino (4%, 44/1009), and Native American or Alaska Native (1%, 9/1009). Additionally, 1% (12/1009) of respondents listed their ethnicity as “other” and 1% (6/1009) preferred not to say. When asked, 71% (717/1009) of respondents were not at high risk for severe COVID-19 illness, 27% (274/1009) were at high risk, and 2% (18/1009) preferred not to say.

Based on the number of respondents reported to be living in their household, they were asked about their household income. The majority of the respondents (58%, 581/1,007) reported being at the highest income level listed (above 250% of the federal poverty level), 30% (300/1,007) were in the middle-income level listed (between 138 and 250% of the federal poverty level) and 13% (126/1,007) were in the lowest income level listed (below 138% of the federal poverty level). The majority of respondents (86%, 865/1,008) had not lost their employment at the time the survey was distributed, while 12% (125/1008) reported being unemployed and 2% (18/1,008) preferred not to say. Most (79%, 792/1,009) respondents had health insurance during the entire study period, 5% (48/1,009) had health insurance for part of the study period, 15% (147/1,009) did not have health insurance during the study period and 2% (22/1009) preferred not to say. In the remaining analysis we additionally assessed responses in vulnerable vs. non-vulnerable respondents. People were considered vulnerable if they met any of the following criteria: self-identified as high risk for severe COVID-19 illness based on CDC definition as of June 4, 2020, household income fell below 250% of the federal poverty level, race/ethnicity was reported as either Black/African American, Hispanic/Latino, or Native American, employment was lost during the pandemic, respondent did not have health insurance, or only had it during part of the study period and respondents who reported that inability to use public transportation or inability to access a car made it more challenging to go to the veterinarian during the pandemic, under this definition, 71% (716/1,009) of respondents were categorized as vulnerable.

#### Impact of the Pandemic on Owners' Ability to Care for Their Pets

Most pet owners (72%, 717/1,003) indicated that their pet needed routine wellness care during the study period, while 38% (376/1,003) reported that their pets needed sick/emergency care, and 9% (91/1,003) reported the need for elective surgery. Of those who wrote in an answer (3%, 31/1,003), 26% (8/31) said their animal needed to be humanely euthanized and 23% (7/31) said they needed a medication consult or refill. When their pet needed veterinary care during the pandemic, 87%, (869/1,004) of respondents indicated that their pet received care from a veterinarian, while 13% (126/1,004) indicated that their pet did not receive care and 1% (9/1,004) preferred not to say. There was no statistically significant difference (Chi square test; *p* > 0.05) in the frequency of pets of non-vulnerable pet owners receiving veterinary care vs. the pets of vulnerable pet owners.

**Figure 1:**
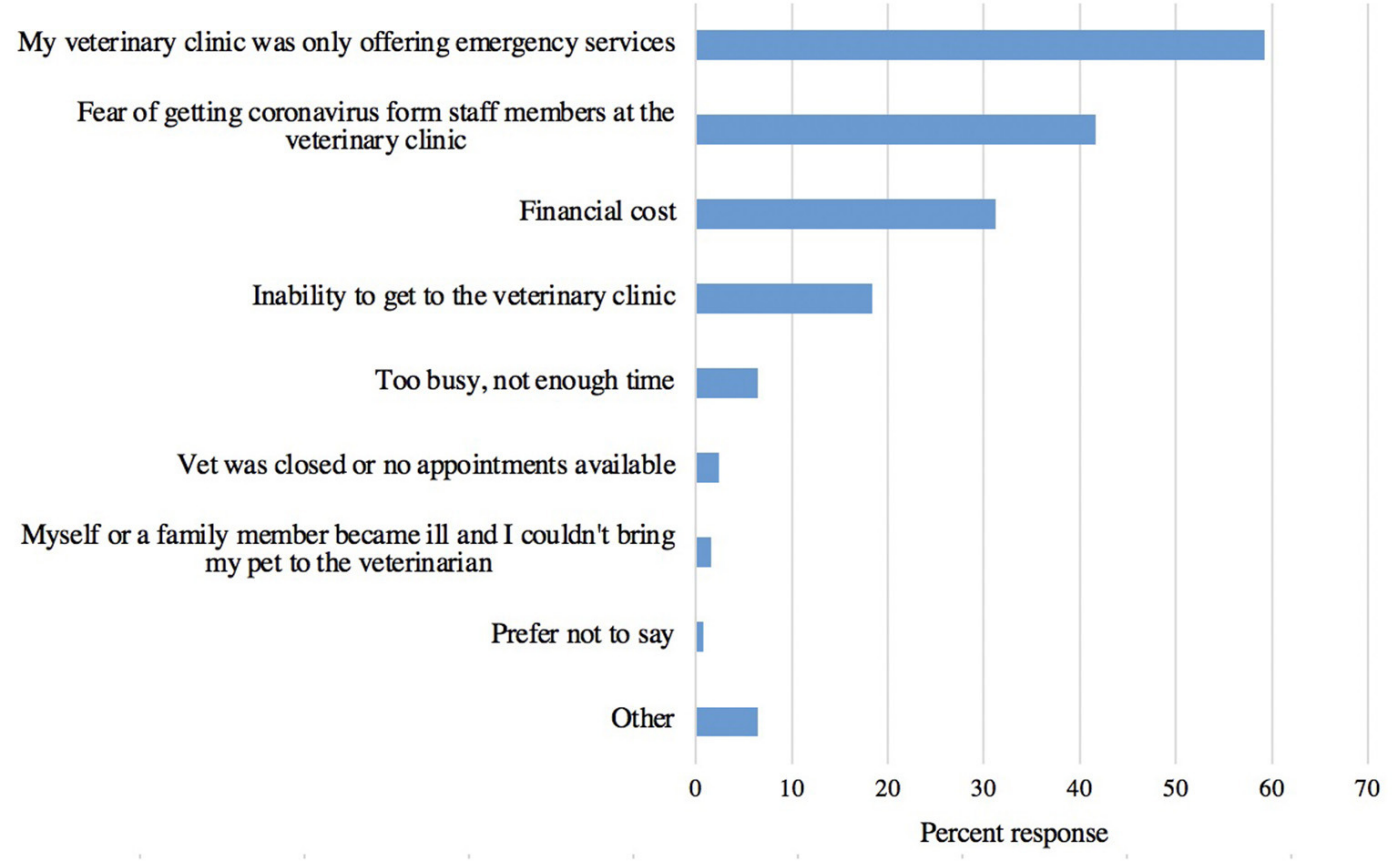
The frequency of reasons responding pet owners selected for why they did not seek veterinary care during the pandemic when their pet needed it (*n* = 125).

The top three reasons for which owners did not seek veterinary care when their pet needed it were that their veterinarian was only offering emergency services, they feared getting coronavirus from staff members, and financial cost barriers (Figure 1). Table 1 presents the breakdown of the frequency of these reasons in non-vulnerable pet owners compared to vulnerable pet owners. While the top three reasons are in the same order between the two groups, a greater percent of vulnerable pet owners (41%, 33/80) selected financial cost compared to the 13% (5/38) of non-vulnerable pet owners, a difference that was statistically significant (Chi-square test; *p* = 0.0045). A veterinary clinic only offering emergency services was ranked consistently as the number one reason to not pursue veterinary care for both groups, however, a greater percent of non-vulnerable pet owners (74%, 28/38) selected this option compared to the 56% (45/80) of vulnerable pet owners, though this value was not statistically significant (Chi-square test; *p* > 0.05). Additionally, 26% (21/80) of vulnerable pet owners indicated that an inability to get to the veterinary clinic prevented them from pursuing veterinary care, compared to 2% (1/38) of non-vulnerable pet owners, a difference that was statistically significant (Chi-square test; *p* = 0.0047).

**Table 1:** Ranking of reasons why non-vulnerable and vulnerable respondents did not pursue veterinary care during the pandemic.

	**Not vulnerable (n = 38)**	**Vulnerable (n = 80)**
1	My veterinary clinic was only offering emergency services (74%)	My veterinary clinic was only offering emergency services (56%)
2	Fear of getting coronavirus from staff members at the veterinary clinic (47%)	Fear of getting coronavirus from staff members at the veterinary clinic (43%)
3	Financial Cost (13%)	Financial Cost (41%)
4	Too busy, not enough time (11%)	Inability to get to the veterinary clinic (26%)
5	Inability to get to the veterinary clinic (3%)	Too busy, not enough time (5%)
6	Myself or a family member became ill, and I couldn't bring my pet to the veterinarian (0%)	Myself or a family member became ill, and I couldn't bring my pet to the veterinarian (3%)

**Figure 2:**
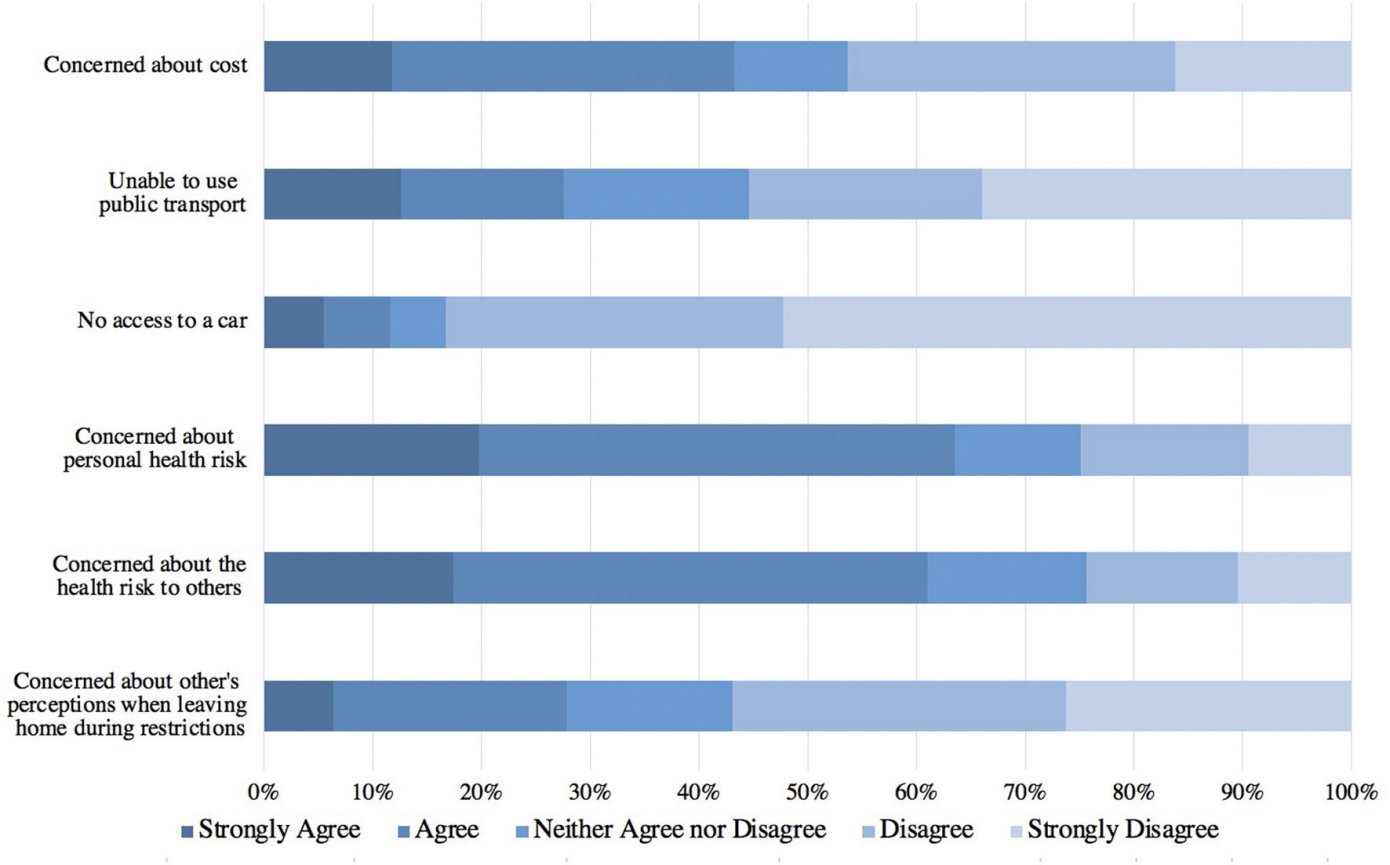
The frequency of reasons responding pet owners gave when asked about what factors made it challenging for the surveyed pet owners to take their pet to the veterinarian during the pandemic (*n* = 998–1,003).

The majority of respondents either agreed or strongly agreed that concerns about their personal health risk (64%, 635/1,000) and the health risk to others (61%, 611/1,001) made it more challenging for them to take their pet to the veterinarian during the pandemic (Figure 2). A minority of respondents agreed or strongly agreed that the inability to utilize public transport, the lack of access to a car, and concerns about how others would perceive them if they left their home during restrictions made it more challenging to take their pet to the veterinarian during the pandemic. Concerns about the financial cost of veterinary care was more evenly split, with 47% (465/1,003) indicating disagreement or strong disagreement and 43% (434/1,003) indicating agreement or strong agreement.

**Figure 3:**
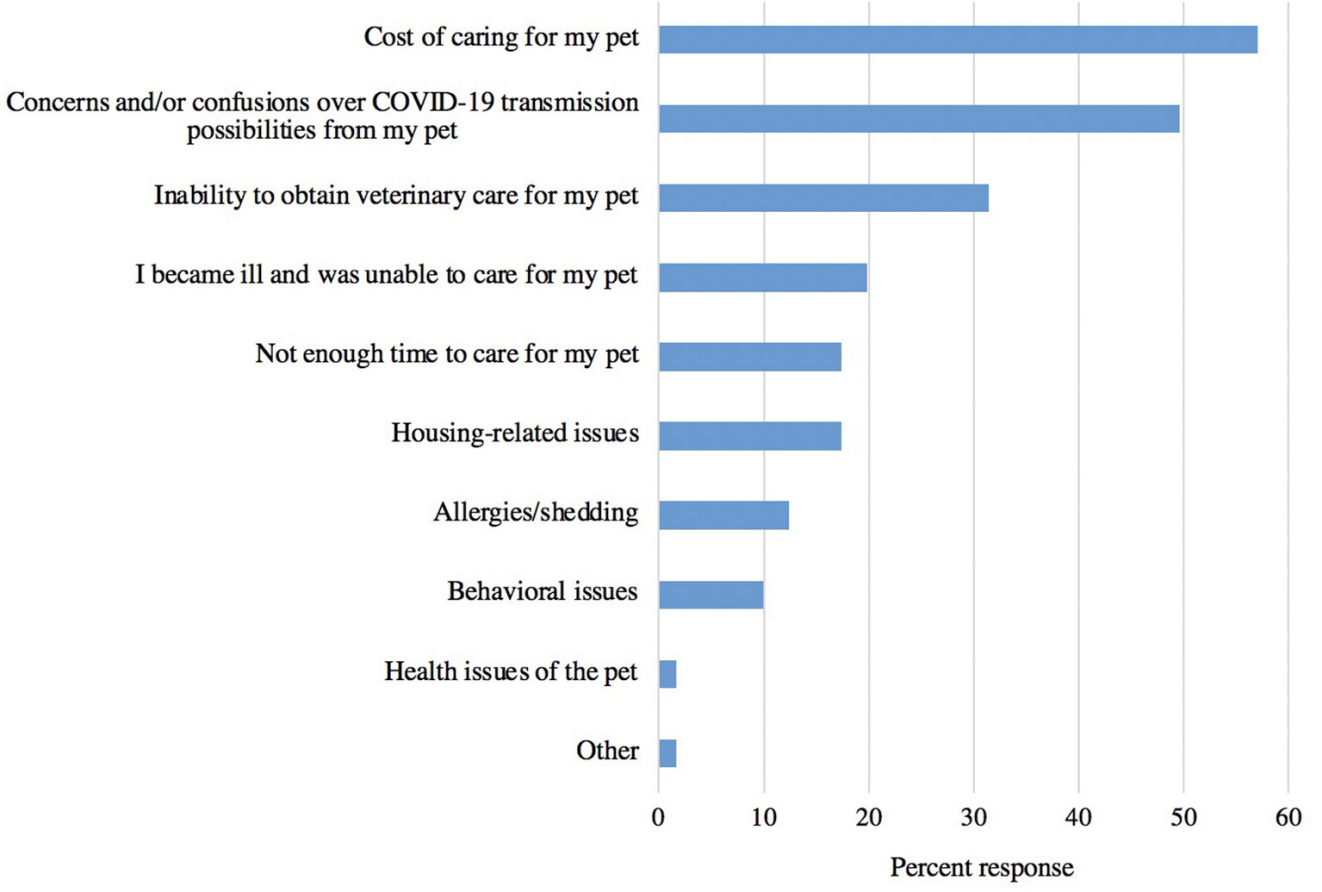
The frequency of reasons responding pet owners considered surrendering their pet during the pandemic (*n* = 121).

Only 12% (121/1,003) of respondents considered surrendering their pet during the study period while 87% (877/1,003) did not and 1% (5/1,003) preferred not to say. Of the 12% of owners who considered it, 42% (51/121) did end up surrendering their pets. Of the owners who considered it, the most frequently reported reason was the cost of caring for the pet, followed by concerns/confusion over COVID-19 transmission possibilities, and inability to obtain veterinary care for the pet (Figure 3). Vulnerable pet owners were significantly more likely to consider surrendering their pets than non-vulnerable pet owners (Chi-square test; *p* < 0.0001).

#### Owner Perceptions

Respondents were asked their level of agreement with a series of statements about their pet during the study period. An overwhelming majority of owners either agreed (37%, 366/1,001) or strongly agreed (57%, 568/1,001) that their pet cheered them up during the pandemic, while fewer disagreed (1%, 14/1,001), strongly disagreed (1%, 5/1,001), or felt neutral (5%, 48/1,001). The majority also agreed (40%, 401/1,000) or strongly agreed (42%, 418/1,000) that their pet gave them purpose during the pandemic, while fewer disagreed (4%, 38/1,000), strongly disagreed (1%, 8/1,000), or felt neutral (14%, 135/1,000). Lastly, the majority of respondents agreed (41%, 406/1,002) or strongly agreed (42%, 425/1,002) that their pet kept them active during the pandemic, while fewer disagreed (6%, 57/1002), strongly disagreed (1%, 14/1,002), or felt neutral (10%, 100/1002).

When asked their level of agreement with statements about disease transmission, the majority of respondents either agreed (30%, 301/1,001) or strongly agreed (52%, 516/,1001) that they never worried about getting coronavirus from their pet, while fewer disagreed (7%, 72/1,001), strongly disagreed (2%, 21/1,001) or felt neutral (9%, 91/1,001). The majority also agreed (41%, 414/1,001) or strongly agreed (47%, 474/1,001) that they trust their veterinarian to give them information on zoonotic diseases, while fewer disagreed (2%, 19/1,001), strongly disagreed (1%, 9/1,001) or felt neutral (8%, 85/1,001). Lastly, the majority of respondents either agreed (38%, 385/1,002) or strongly agreed (20%, 204/1,002) that they were comfortable with the idea of going to the vet during the pandemic, while fewer disagreed (18%, 177/1,002), strongly disagreed (4%, 42/1,002), or felt neutral (19%, 194/1,002).

#### Telemedicine

When asked if they had ever utilized telemedicine platforms for their veterinary care, most respondents (73%, 736/1,002) said they never had, 7% (67/1,002) said they had used telemedicine prior to the pandemic, 14% (143/1002) said they used it exclusively during the pandemic, and 4% (36/1,002) said they used it both before and during the pandemic. Only ~1% (13/1,002) of respondents said they were not sure and ~1% (7/1,002) preferred not to say. Vulnerable pet owners were statistically more likely to have used telemedicine compared to non-vulnerable pet owners (Chi-square test; *p* < 0.0001).

Owners who reported having used telemedicine during the study period were asked to select all reasons for which they did so. The most frequently indicated reasons were that their veterinarian recommended it (58%, 104/178), that telemedicine was easier than going into the veterinary clinic (55%, 98/178), and that respondents were worried about their risk of getting coronavirus by going to the veterinary clinic (47%, 83/178). Less commonly selected answers included that telemedicine was the only option because the veterinarian was only open for emergencies (29%, 52/178), and that they had used telemedicine prior to the pandemic (10%, 17/178). A majority (84%, 124/147) of respondents who used telemedicine during the pandemic said that they would have physically brought their pet to the veterinarian to receive care during the pandemic if telemedicine had not been an option, 15% (22/147) said that they would not have, and 1% (1/147) preferred not to say. The majority of respondents either agreed (65%, 159/244) or strongly agreed (24%, 58/244) that they were satisfied with the care their pet received remotely through telemedicine. Fewer respondents disagreed (2%, 5/244), strongly disagreed (1%, 2/244), or felt neutral about the statement (8%, 20/244). In addition, the majority of respondents either agreed (50%, 122/244) or strongly agreed (30%, 72/244) that they would be interested in using telemedicine in the future, while fewer indicated that they disagreed (6%, 15/244), strongly disagreed (1%, 2/244) or felt neutral about the statement (14%, 33/244).

Respondents reporting that they had never used telemedicine to receive veterinary care were asked to select all reasons for which they did not utilize it. The most commonly indicated reason was their veterinarian not offering it or making them aware of it (67%, 492/733). Second to this, owners said that the care their pet needed could not be done with telemedicine (44%, 324/733). Concerns about quality of care versus an in-person appointment was the third most frequently selected reason (27%, 201/733). Far fewer respondents indicated that they did not have the technology to access it (2%, 16/733) or that the technology seemed too difficult to use (2%, 11/733). Of the 1% (7/733) of respondents who wrote in an answer, 71% (5/7) of them said that they didn't use telemedicine because it was unnecessary, as their vet was doing all services in person. Finally, 67% (389/579) of respondents who had never used telemedicine before indicated that they would be interested in using telemedicine in the future, while 33% (190/579) said that they would not be interested.

### Veterinary Clinic Survey

#### Demographics

A total of 516 veterinary clinic staff participated in the survey. The majority (63%, 327/516) of respondents were veterinarians (42%, 218/516 associate veterinarians, 16%, 85/516 owning veterinarians, and 5%, 24/516 classified as other). Veterinary technicians made up 24% (124/516) of respondents. The remainder worked in reception or client services (2%, 12/516), were office managers (3%, 18/516), or classified as “other” (7%, 35/516). Respondents primarily worked at privately owned (53%, 271/513) or corporate owned (35%, 179/513) practices, followed by academia/teaching hospitals (4%, 21/513), publicly owned clinics (3%, 15/513), non-profits (2%, 11/513), and shelters/rescues (1%, 4/513). The ownership status of their clinic was classified as “other” by 2% (12/513) of respondents.

Additional demographics included age; 32% (165/516) of respondents were 30–39 years old, 23% (119/516) were 40–49 years old, 20% (101/516) were less than 30 years old, 17% (85/516) were 50–59 years old, 7% (37/516) were 60–69 years old, and 2% (9/516) were greater than 70 years old. The majority of respondents identified as female (85%, 439/516), followed by male (13%, 68/516) and prefer not to say (2%, 9/516). Respondents were asked to identify the state they live in, and then the regional definitions put forward by the U.S. Census Bureau were used to group respondents into four regions: Northeast, South, Midwest, and West. The West had the most respondents (32%, 160/492), followed by the South (28%, 138/492), the Northeast (23%, 112/492), and the Midwest (17%, 82/492). The length of time respondents worked in veterinary medicine varied from >20 years (30%, 146/484), 5–10 years (25%, 120/484), <5 years (19%, 94/484), 11–15 years (16%, 76/484) and 16–20 years (10%, 48/484). Most respondents practiced in suburban areas (54%, 275/513), followed by urban areas (27%, 139/513), and rural areas (18%, 91/513), while 1% (4/513) of respondents practiced in areas they defined as a mix of the answer options, and 1% (4/513) listed “other.” The vast majority of respondents practiced small animal predominant medicine (93%, 476/513). Less frequently selected were large animal predominant (3%, 14/513), unspecified academia practice (2%, 10/513), exotics or zoo (1%, 7/513), and other (1%, 6/513).

#### Impact of the Pandemic on Providing Care to Traditionally Vulnerable Populations

**Figure 4:**
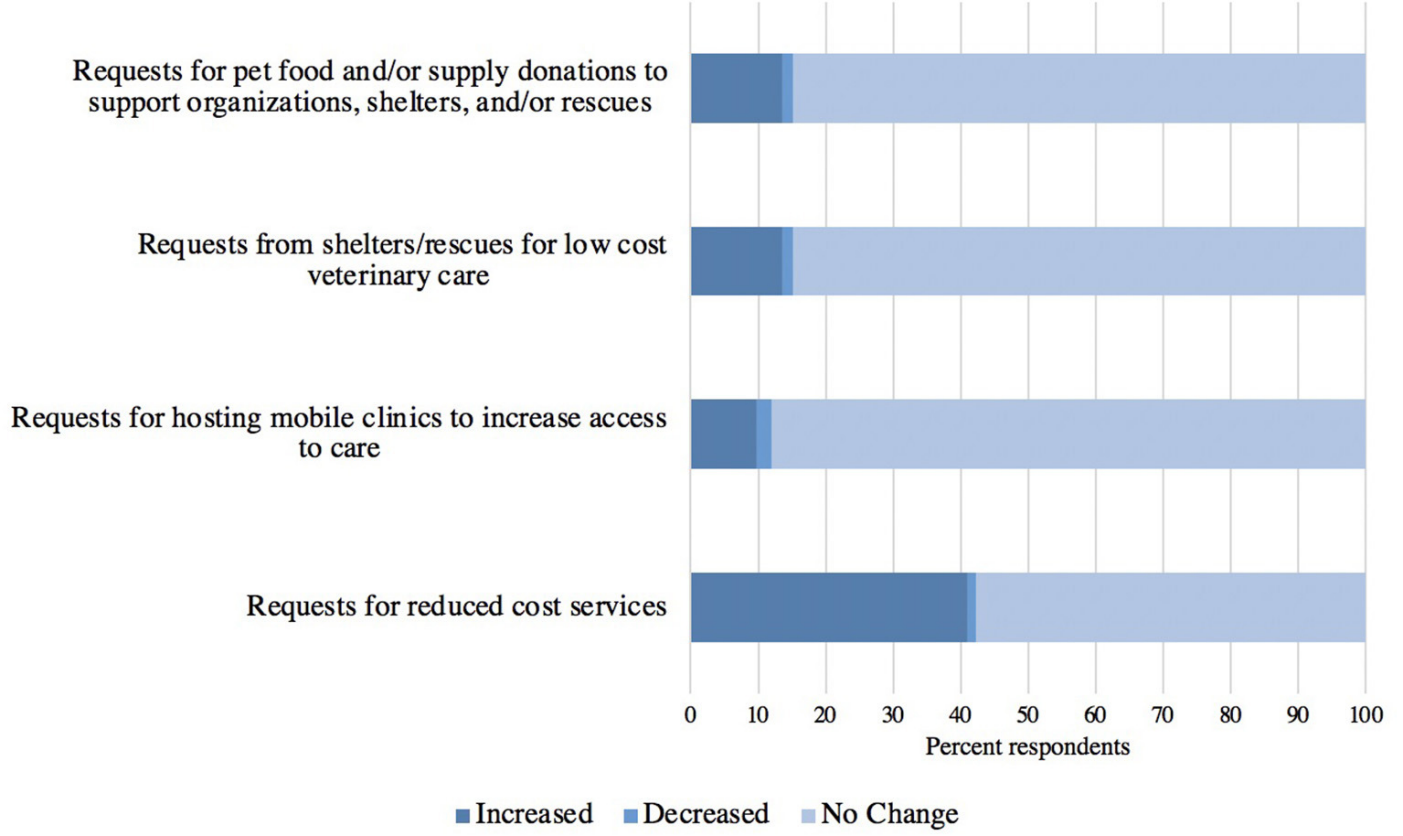
The frequency in which responding veterinary practices received various requests from pet owners and outside organizations related to vulnerable populations during the pandemic (*n* = 481–499).

**Figure 5:**
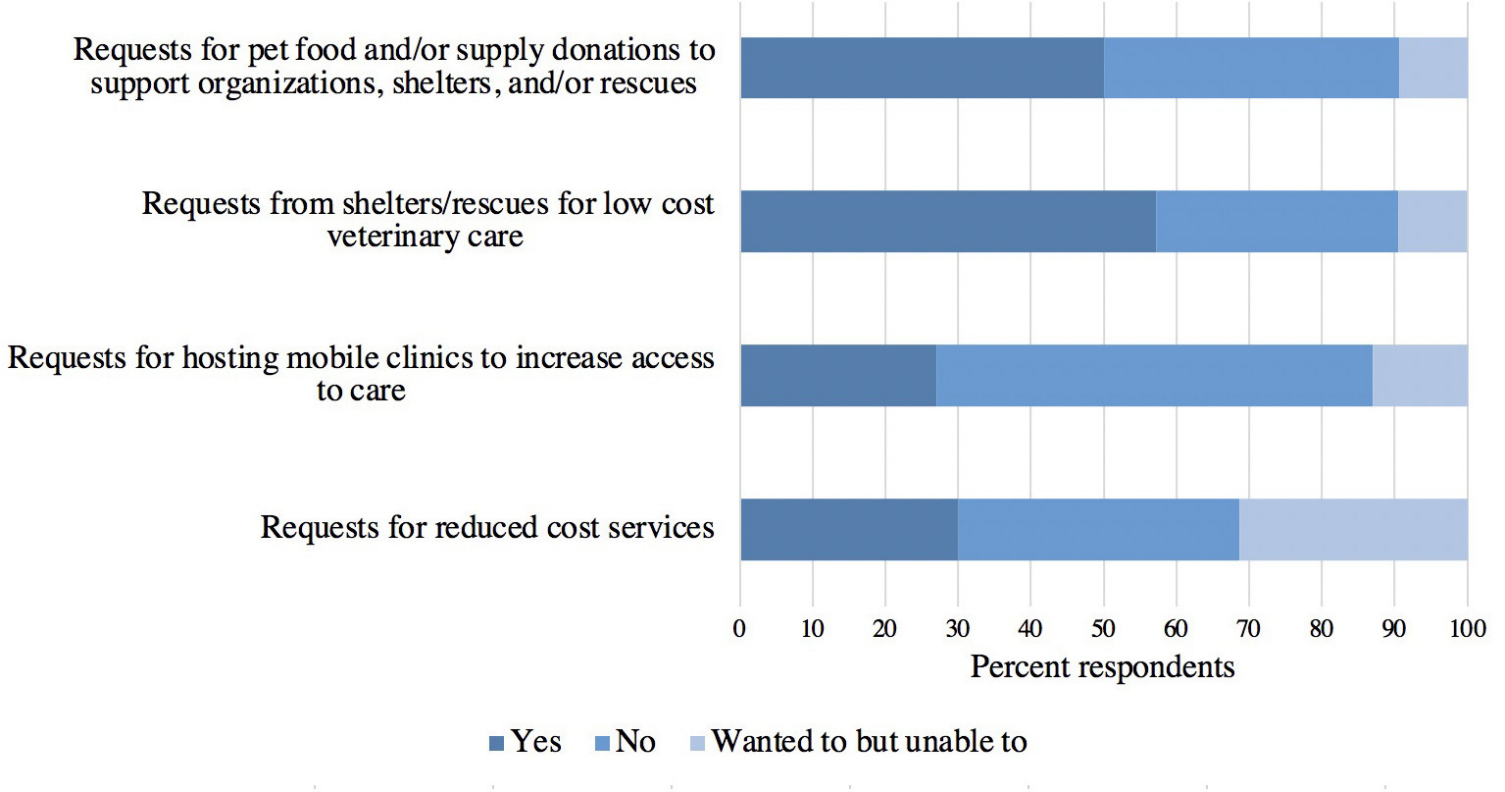
Ability of responding veterinary clinics to accommodate received requests from pet owners and outside organizations for various low-cost services or products (*n* = 427–435).

The frequency in which practices received various requests related to vulnerable populations is presented in Figure 4. Requests for reduced cost services increased the most (41%, 204/499), while the other requests increased by less than 20%. Figure 5 shows the responses when asked about their practice's ability to respond to these requests. Of those that indicated their practice received an increase in the request for low-cost services, the majority responded that they were able to accommodate requests to support shelters and rescues (Figure 5), while at the same time the majority of respondents were unable to accommodate requests for reduced cost services that did not originate from shelters and rescues and they were unable to host mobile clinics. When asked if there was anything else respondents would like to share about requests received during the pandemic, 21% (20/97) of those who answered expressed that they did not receive any of the pandemic-related requests presented in the questionnaire.

Respondents were asked to select any resources that would best help them to increase their role in supporting vulnerable populations. The most frequently selected option was resources on how to create a sustainable system for their practice to support clients in financial need (53%, 258/490). This was followed by resources on how their veterinary practice can play an active role in companion animal care for vulnerable populations (33%, 164/490), resources on a veterinarian's role in public health for vulnerable populations (32%, 158/490), instruction in vet school on companion animal care for vulnerable populations (32%, 158/490), and resources on how to approach and interact with vulnerable populations (29%, 144/490). Other notable answers that were written in include resources on how to encourage a clinic-culture of non-judgement and decreased resentment, information on organizations who are looking for veterinarians to help vulnerable populations, and resources to improve community partnerships for care that cannot be provided by the practice.

#### Impact of the Pandemic on Providing Care to Newly Vulnerable Clients

**Figure 6:**
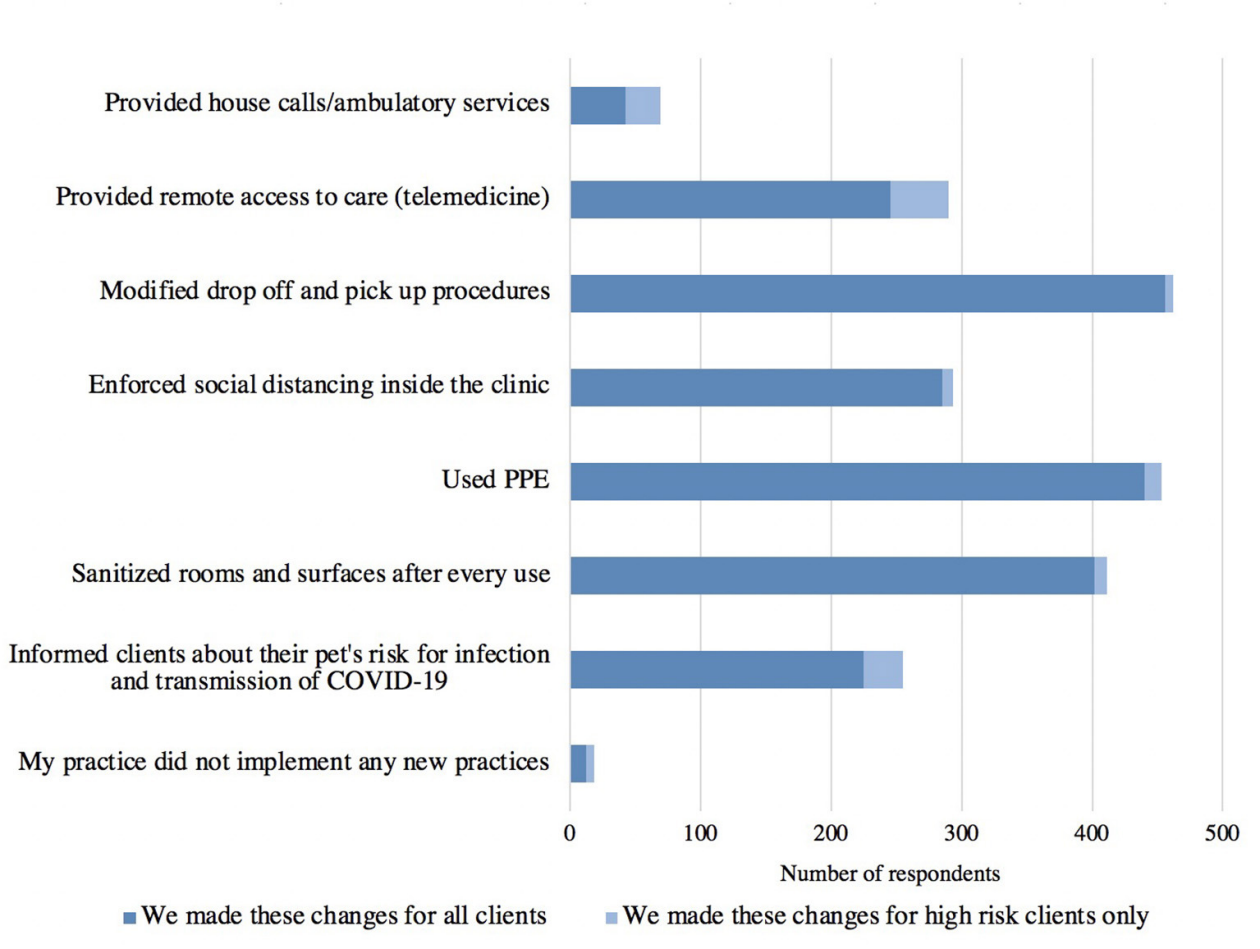
Changes responding veterinary practices made to support all and high-risk clients during the pandemic (*n* = 516).

**Figure 7:**
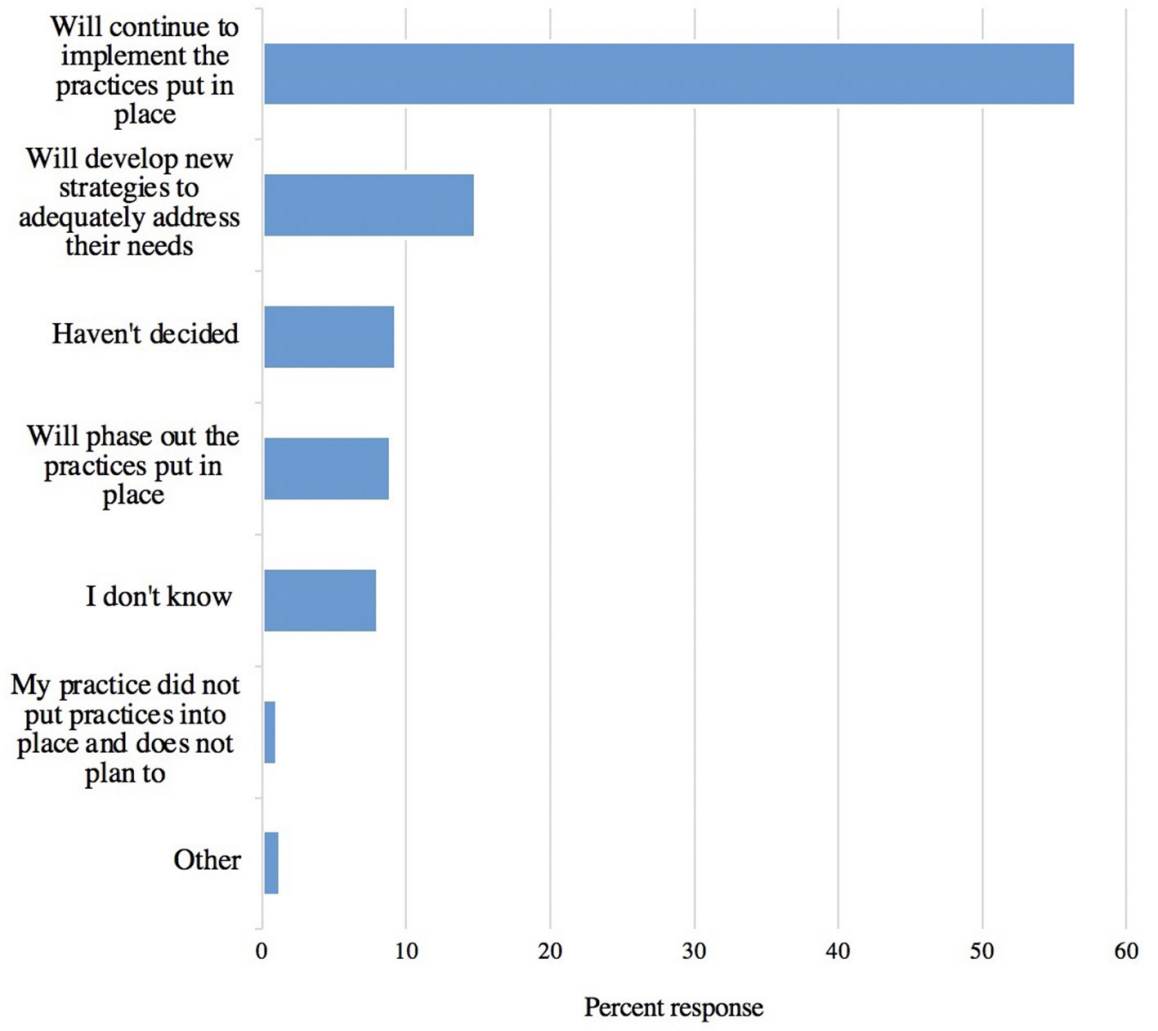
Ways in which responding veterinary practices planned to move forward as pandemic recovery continues at the time of the survey (*n* = 471).

Respondents were asked about changes they made in their practice in order to better support clients during the pandemic (Figure 6). Veterinary practices most frequently implemented modified drop off and pick up procedures, use of personal protective equipment (PPE), and sanitization of rooms and surfaces after every use. Most respondents for each option indicated that the practice was put in place for all clients rather than just for high-risk clients. The use of telemedicine had the highest number of respondents to indicate that it was only instituted for high-risk clients. The majority of respondents (56%, 266/471) indicated that their practice plans to continue to implement the practices they had put in place during the pandemic (Figure 7).

**Figure 8:**
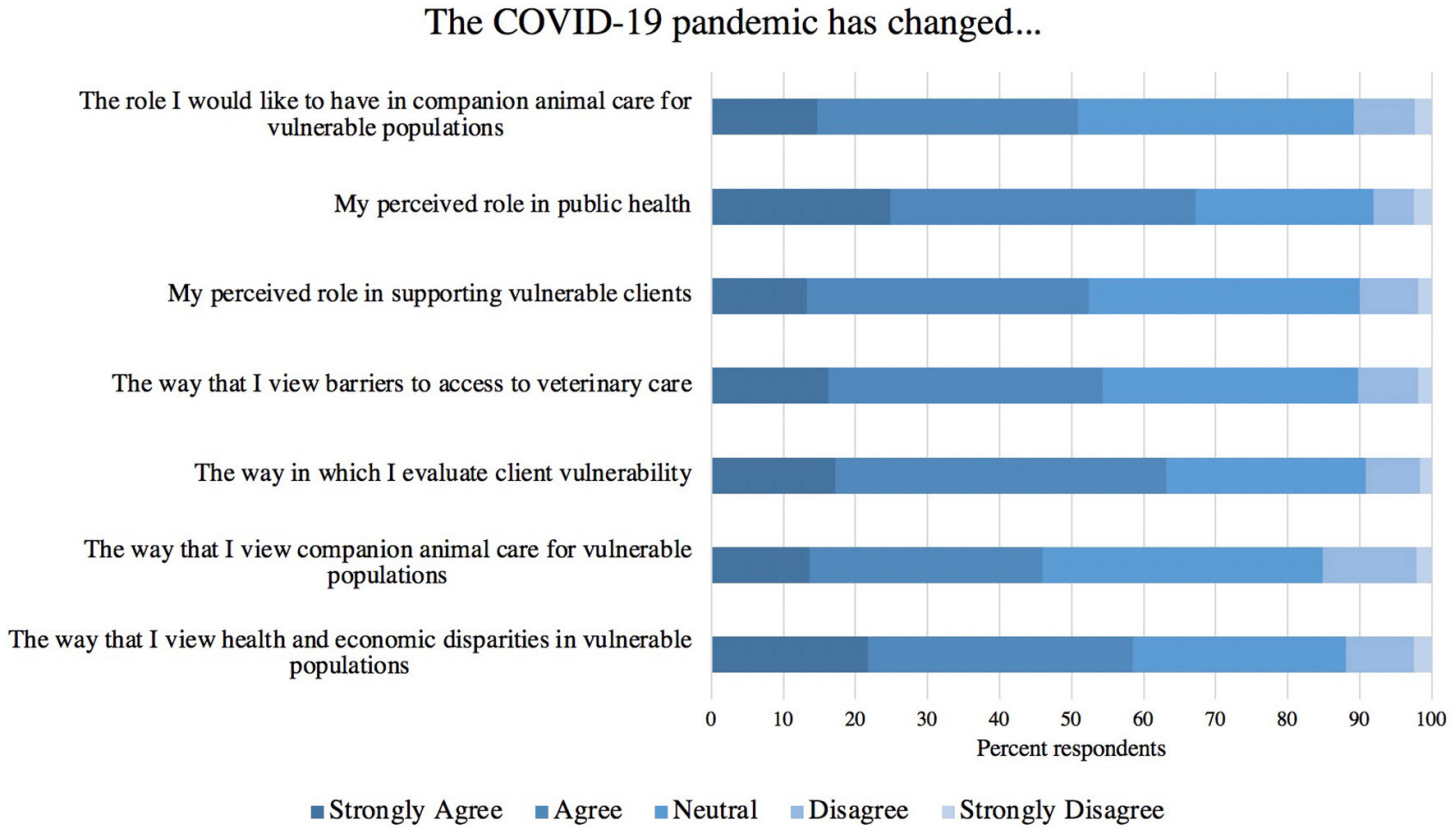
Responding veterinarians' level of agreement with statements assessing the pandemic's influence on views and perceptions of aspects of veterinary medicine and vulnerable populations (*n* = 466–470).

When asked their level of agreement with statements regarding the pandemic's influence on their role as a veterinarian (Figure 8) the largest percent of respondents agreed or strongly agreed that the COVID-19 pandemic has changed their perceived role in public health. The majority of respondents agreed or strongly agreed with all other statements regarding vulnerability except that the COVID-19 pandemic has changed the way that they view companion animal care for vulnerable populations. Respondents were asked about any other perceptions that changed as a result of the pandemic, and of those who answered, the most common answer respondents remarked on was the extent that they believe the profession is undervalued or overlooked (10%, 7/72). Respondents were also asked to share anything else regarding access to care for vulnerable populations or high-risk clients, and the most common answer was the potential of telemedicine to increase access to care, which was discussed by 11% (4/37) of those who answered.

#### Telemedicine

**Figure 9:**
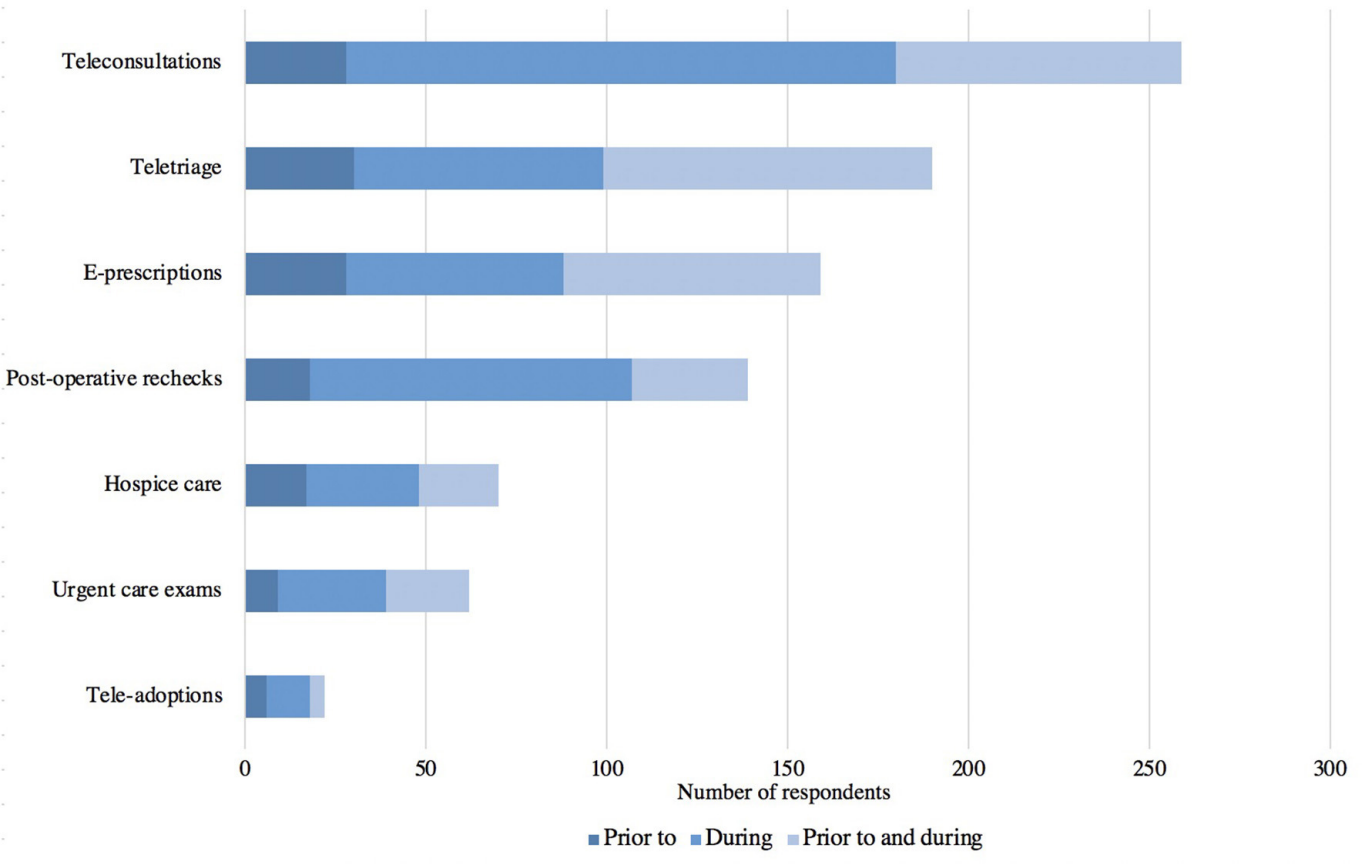
Telehealth or telemedicine services offered by responding veterinary practices and if they used them, prior to, during or prior to and during the pandemic (*n* = 288).

**Figure 10:**
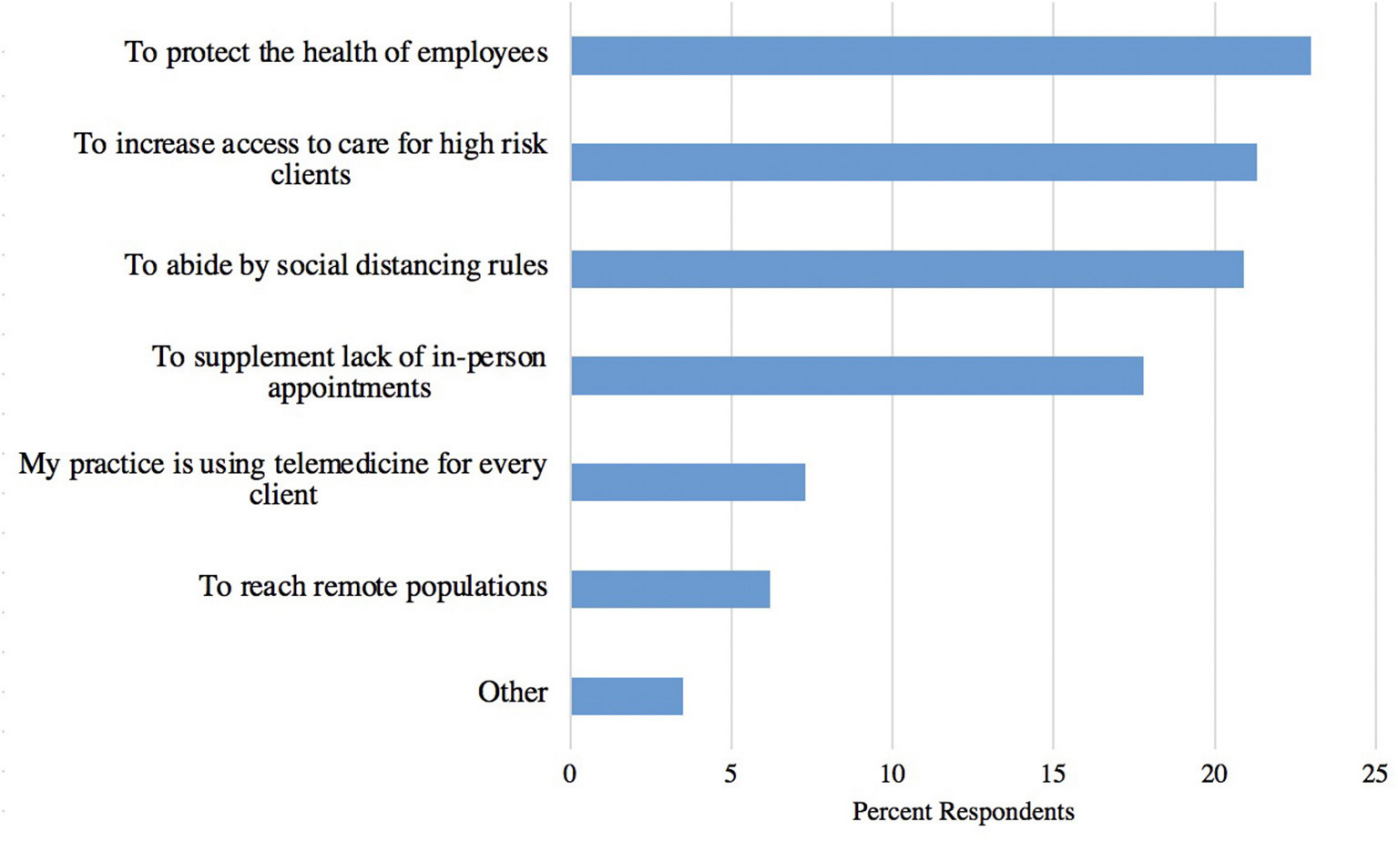
Reasons for which responding veterinary practices implemented telemedicine during the pandemic (*n* = 283).

Most respondents indicated that the pandemic either increased (50%, 230/464) or significantly increased (19%, 88/464) their interest in telemedicine, while fewer indicated their interest decreased (5%, 22/464), or significantly decreased (1%, 3/464), and 26% (121/464) of respondents said the pandemic did not change their opinion of telemedicine. Most respondents (63%, 288/456) used telemedicine during the pandemic, while 37% (168/456) did not. The types of telemedicine utilized prior to, during, and prior to and during the pandemic are shown in Figure 9. Teleconsultations were the most frequently utilized, followed by teletriage and E-prescriptions. The three most frequently selected reasons for implementing telemedicine were to protect the health of employees, to increase access to care for high-risk clients, and to abide by social distancing rules (Figure 10). Of the respondents who wrote in an answer for reasons they used telemedicine, 19% (5/27) indicated that it was because their clients requested it, and 15% (4/27) said that it was what they were doing prior to the pandemic.

Of the respondents whose clinic had utilized telemedicine, very few (5%, 11/205) indicated that they would not continue using telemedicine to provide veterinary care for their clients as pandemic recovery continues, while 70% (143/205) indicated that they would continue the use of telemedicine for all clients, and 25% (51/205) said they would continue it for clients at high risk for severe COVID-19 illness. Of the respondents who wrote-in additional thoughts on telemedicine, 16% (13/83) expressed concerns over standard of care, misdiagnosis, and the inability to perform a physical examination. Additionally, 12% (10/83) discussed their positive experiences in telemedicine and their belief in its use in the profession.

The majority of veterinarians and veterinary technicians said that they did not learn about telemedicine in their veterinary medicine curriculum (90%, 327/365), while 10% (38/365) indicated that they did. When asked if they thought veterinary medical school should make changes to curriculum content in response to the pandemic, 75% (276/370) of all respondents answered yes, while 25% (94/370) answered no. Those who answered yes were then asked the degree to which they agreed or disagreed with the implementation of several topics to the veterinary curriculum. Most respondents either agreed (54%, 149/274) or strongly agreed (41%, 111/274) that instruction on telemedicine use should be added to the curriculum, while fewer disagreed (0.4%, 1/274), or felt neutral (5%, 13/274). Most respondents also agreed (35%, 95/275) or strongly agreed (53%, 146/275) that there should be increased instruction on public health and zoonotic disease, while fewer disagreed (1%, 3/275), or felt neutral (11%, 31/275). Lastly, most respondents either agreed (50%, 136/274) or strongly agreed (41%, 112/274) that there should be instruction on vulnerabilities and access to care, while fewer disagreed (0.4%, 1/274), or felt neutral (9%, 25/274). No respondents strongly disagreed with the implementation of any of these topics to the veterinary curriculum.

### Interviews With Access to Care Organizations

#### Barriers to Care and Pandemic Services

**Figure 11:**
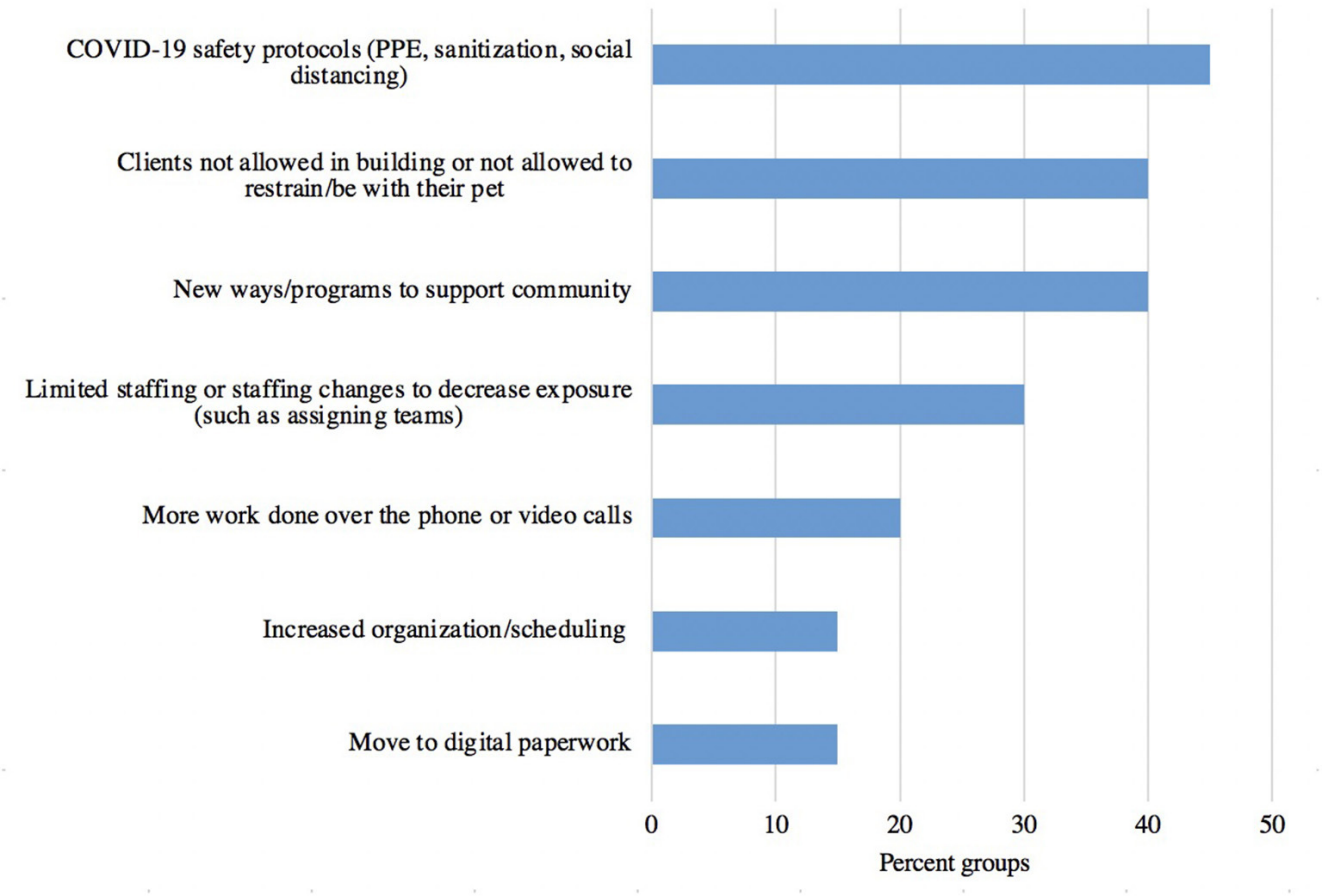
Strategies and procedural changes responding access to care organizations used to adapt their operations to support vulnerable communities during the pandemic (*n* = 20).

Prior to the onset of the pandemic, most (80%, 16/20) of the access to care organizations represented in the interviews reported to provide preventative care and/or spay and neuters. Pre-pandemic 45% (9/20) of the interviewed organizations provided sick and emergency veterinary care, and 20% (4/20) provided non-veterinary animal-related services, including animal assisted therapy, grooming and provision of supplies. Of the 20 access to care organizations represented, 80% (16/20) stopped all normal services at the onset of the pandemic. At the time of the interviews (July 24–September 27, 2020) 38% (6/16) of those organizations had not resumed any normal services, 31% (5/16) had resumed with limited services or fewer clinics and another 31% (5/16) had resumed with full services, 15% (3/20) of groups never fully stopped providing services, but did scale back at first and then resumed normal services. The most common reason for groups stopping or restricting services was due to government mandates, which was cited by 70% (14/20) of groups, followed by health and safety decisions of the organization or parent organization, cited by 60% (12/20) of groups, and 15% (3/20) of groups mentioned travel restrictions put in place by the communities being served. Figure 11 presents ways in which organizations adapted operations to support communities during the pandemic. The three major themes included implementing COVID-19 safety protocols (45%, 9/20), implementing new ways or programs to support communities (40%, 8/20), and not allowing clients into buildings or not allowing clients to restrain/be next to their pet (40%, 8/20).

Interviewees were asked what barriers to access to veterinary care the communities they served experienced. The most reported barrier was financial (80%, 16/20) followed by transportation (55%, 11/20), geographic (40%, 8/20), and language (25%, 5/20). Judgement or distrust of veterinarians was noted by 20% (4/20) of groups, as well as lack of access to information, education, or communication. Accessibility to local veterinarians (due to local veterinarians being overwhelmed, or the lack of collars, leashes, carriers) was mentioned by 15% (3/20) of groups, and physical or mental inability to get to a veterinary clinic was mentioned by 10% (2/20).

#### Resources and Support

In the conversations, 35% (6/17) of the interviewees reported government guidelines as a resource used to guide decision making on conducting services during the pandemic. Other answers included collaboration with other organizations (29%, 5/17 of interviewees), information released by the American Veterinary Medical Association (AVMA) or shelter groups (24%, 4/17 of interviewees), communications with the communities they serve (18%, 3/17 of interviewees), CDC recommendations (18%, 3/17 of interviewees), social media or webinars (18%, 3/17 of interviewees) and guidance from the parent organization (12%, 2/17 of interviewees). When asked what support could have been used during the pandemic, 41% (7/17) of the interviewees indicated financial support. Mental health support for staff/volunteers, resources and information on best practices/what similar groups were doing, and PPE/supplies/facility support each were mentioned by 24% (4/17) of interviewees. Another 24% (4/17) of interviewees reported that there was no additional support they could have used.

#### Challenges Experienced and Concerns

**Figure 12:**
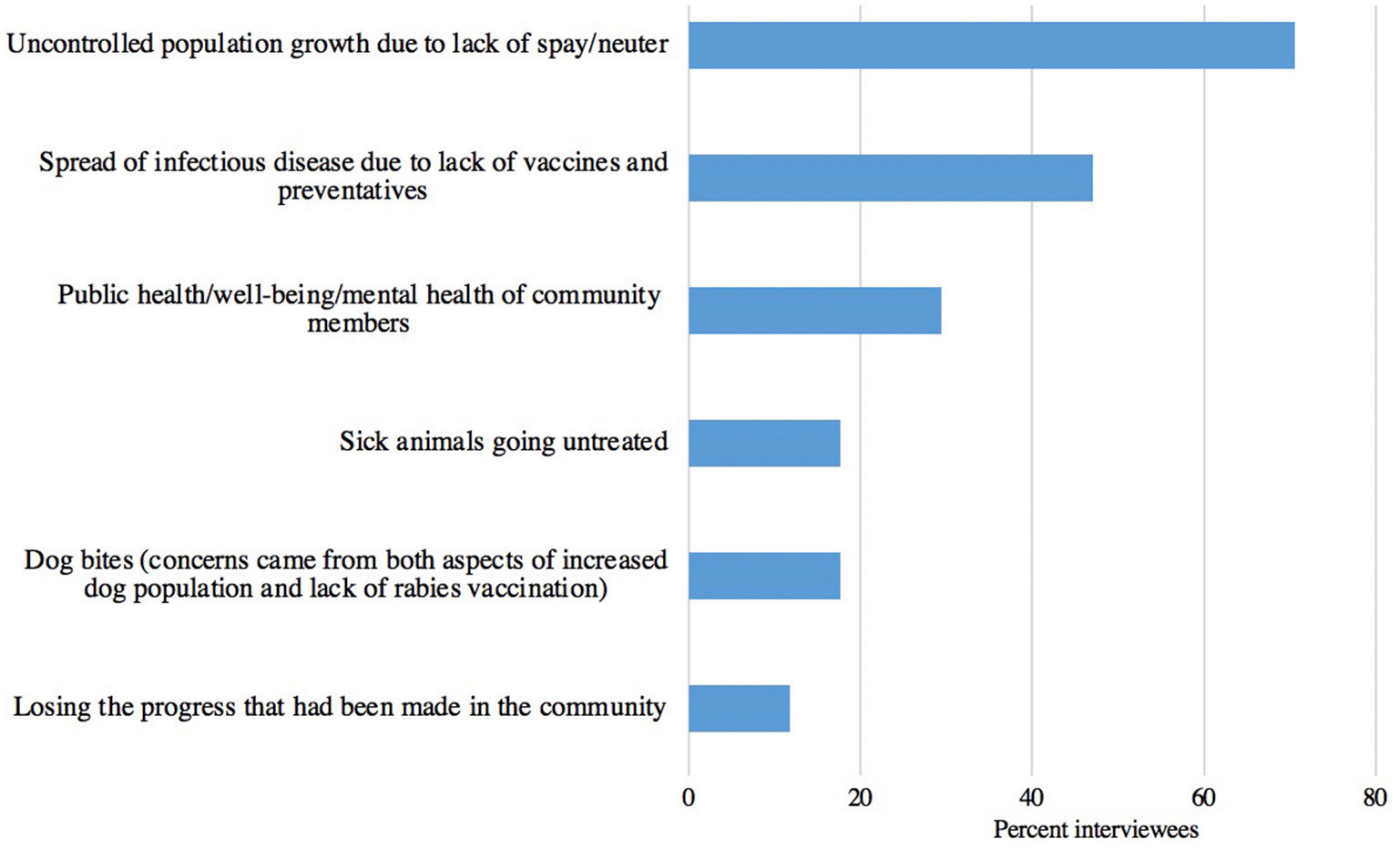
Concerns expressed by access to care organizations about consequences to the community as a result of disruption of veterinary services (*n* = 17).

Figure 12 shows major themes in terms of concerns about consequences in the community as a result of the disruption of services. The most commonly cited concern (71%, 12/17 of interviewees) was about uncontrolled population growth due to the lack of spay and neuter services and 47% (8/17) worried about the spread of infectious disease due to the lack of vaccines and preventatives.

**Figure 13:**
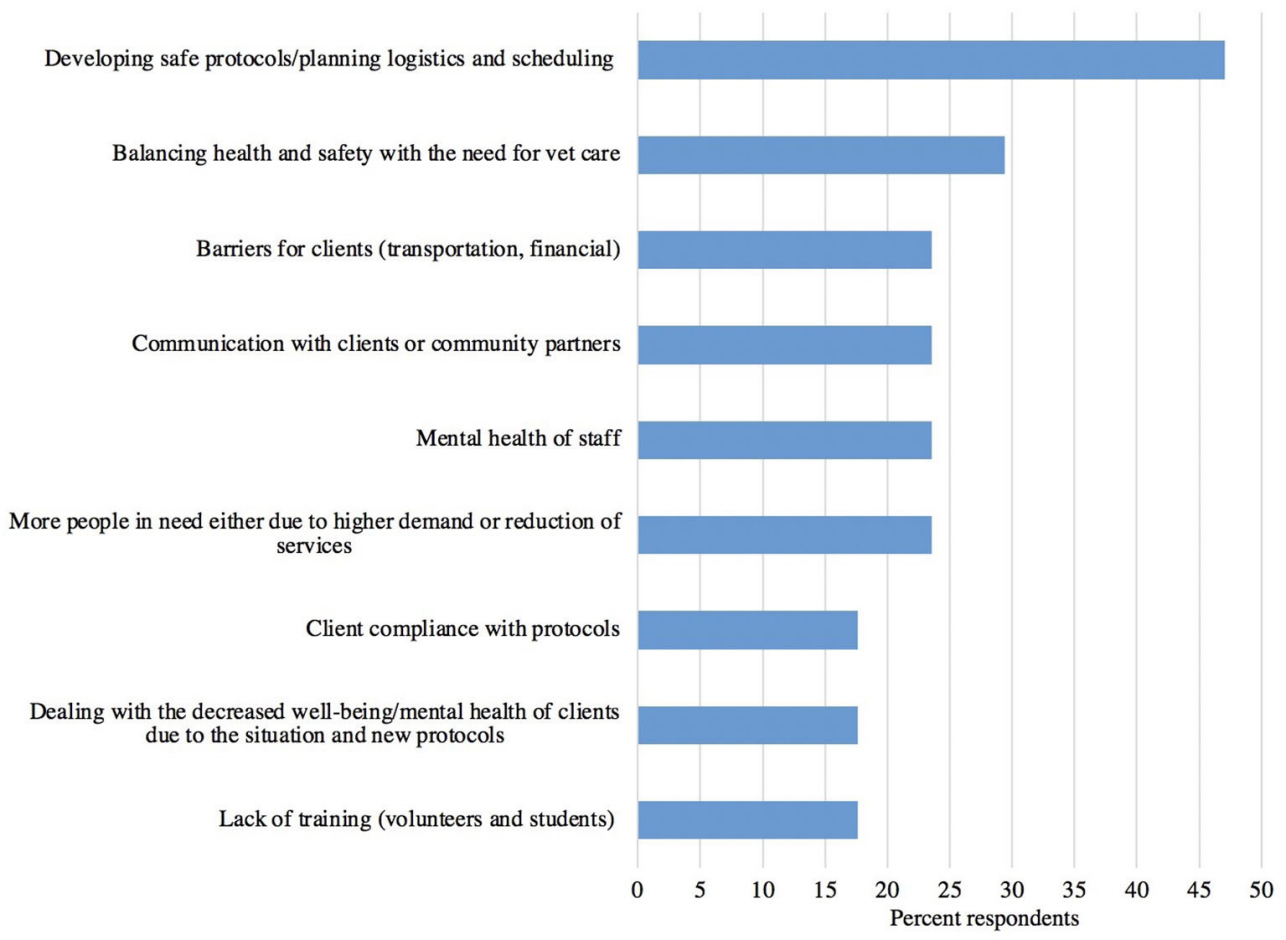
Challenges to providing veterinary services during the pandemic experienced by access to care organizations (*n* = 17).

Two major concerns were noted with respect to resuming services. Most (76%, 13/17) interviewees were concerned with COVID-19 transmission in terms of the health and safety of the communities and staff/volunteers, and 24% (4/17) were concerned about the potential of having to shut down and stop services if a team member were to test positive. Interviewees were asked about challenges to providing services during the pandemic. Major themes are presented in Figure 13, with the challenge of developing safe protocols, planning the logistics, and scheduling being the most commonly mentioned.

When asked about the hardest part of the pandemic, 47% (8/17) of interviewees said the hardest part was not being able to provide normal services. In expressing this sentiment, half of these interviewees elaborated on the mental health toll in knowing that animals and communities were suffering and yet not being able to help like they normally do. Additionally, 35% (6/17) of interviewees mentioned keeping up with the high demand or not being able to help everyone in need, 29% (5/17) said providing services with the new health and safety protocols, 29% (5/17) said missed opportunities for follow up care, collaboration, and spreading the word about the organization, and 18% (3/17) cited financial difficulties.

#### Successes and Opportunities for the Future

When asked about any positive moments during the pandemic, support and appreciation from the communities and the generosity of volunteers, funders, or donors were the most commonly reported themes (35%, 6/17 of interviewees each). Another 24% (4/17) mentioned comradery among organizations/veterinarians, 18% (3/17) of interviewees mentioned comradery within the communities, and 12% (2/17) mentioned comradery and/or adaptability within the team. Increased or new opportunity (18%, 3/17 of interviewees), student engagement (12%, 2/17 of interviewees), and connection with the communities (18%, 3/17 of interviewees) were other themes found. One of the major themes in terms of opportunities that arose as a result of the pandemic was that there became new opportunities to support the communities in unique ways (53%, 9/17 of interviewees). Additionally, 29% (5/17) of interviewees also noted opportunities to reflect on logistics and/or improve efficiency, 18% (3/17) mentioned new ways for community outreach and education, and 12% (2/17) said opportunities to support local partners and veterinarians.

When asked if there were any changes made during the pandemic that they would continue to implement moving forward, over half (59%, 10/17) of interviewees said that there were changes that ended up increasing efficiency and/or organization that would be continued. Of the interviewees, 24% (4/17) said that they would continue new services that were introduced during the pandemic, and 24% (4/17) said that the improved communication with the community would continue. Additionally, 12% (2/17) of interviewees said they would continue with the increased cleaning and disinfection.

**Figure 14:**
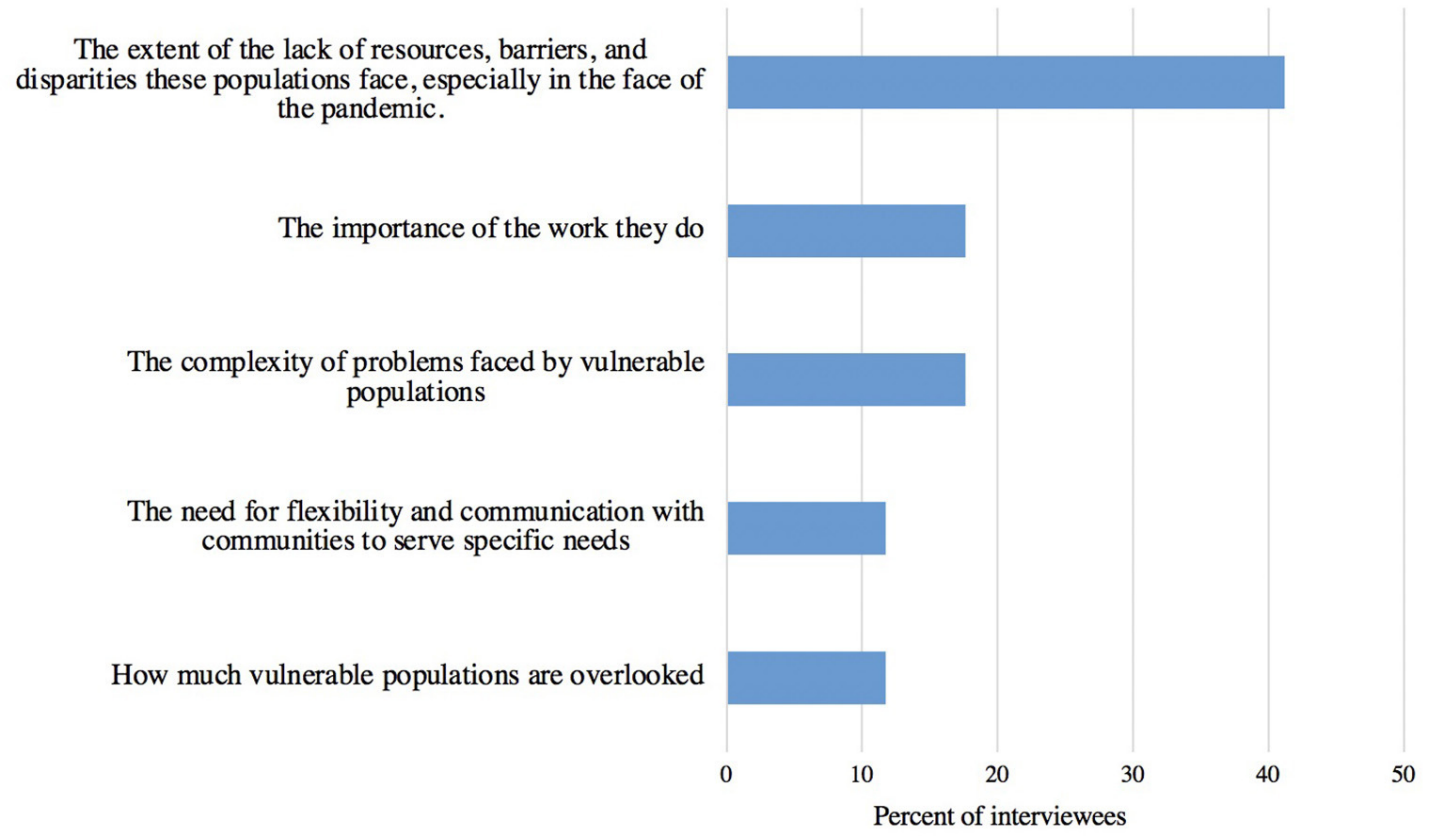
Themes learned by access to care organization interviewees from the pandemic about vulnerable populations (*n* = 17).

Interviewees were asked what they learned during the pandemic about vulnerable populations and/or the support those populations need (Figure 14). The most common themes that emerged was the extent of the barriers, disparities, and lack of resources that the populations face, especially in the face of the pandemic (41%, 7/17 of interviewees).

## Discussion

Collectively, this work highlights how the COVID-19 pandemic created new (ex. heightened infectious disease risk, reduced veterinary services), and exacerbated existing (ex. financial barriers), issues with accessing and providing veterinary care. The pandemic expanded the definition of vulnerability in the human population and the impacts further exacerbated the financial barrier in access to animal welfare and health, as demonstrated by the increased number of requests to veterinary clinics for reduced cost services, and by the disruption (financially and logistically) of low or no cost veterinary services provided by access to care organizations to traditionally vulnerable populations. Society and the veterinary profession have become more aware of the complexity of health and access to care, and the pandemic highlighted the veterinarian's perceived role in public health. Additionally, this study identified areas on which the veterinary profession can focus in order to help increase access to veterinary care, including integration of critical instruction into the veterinary school curriculum and continuing education, and through strategic use of telemedicine.

While this research elucidated interesting information about the response by veterinary clinics, pet owners and access to care organizations during the initial phase of the COVID-19 pandemic, we focus here on broad themes that can help the veterinary profession and access to care organizations be the most prepared to respond when the next disaster or large response effort is needed. These are (1) opportunities for further learning for the veterinary profession; including curricula around telemedicine, financially resilient business models and understanding health disparities and vulnerable populations; (2) a need for a network of collaboration and communication across veterinary clinics and access to care organizations and (3) future preparedness for health, economic or other crises response.

### Opportunities to Expand Education of Service Providers

In order to respond to the current stressors resulting from the pandemic, such as increased requests for reduced cost services, and prepare for future changes in the veterinary field, it is essential to provide veterinarians and students with the necessary background information to be successful. A goal of these educational materials is to prepare students for practice within and beyond traditional clinical practice and to provide a set of resources for creating systems that address barriers to care that can be implemented in practice settings and through access to care organizations and programs. The research presented here shows that the vast majority of veterinary professionals believe that veterinary schools should make changes to their curricula in response to the pandemic, including instruction on telemedicine and access to care. Other studies have shown support for the inclusion of additional topics, such as training on how to educate clients on pet insurance and future veterinary costs (22) as well as training on working with clients who have financial and emotional stress (23). There are several educational methods that should be developed and/or deployed to prepare veterinarians for success, including inclusion of materials in veterinary school curriculums and development of continuing education lectures and focused workshops for practicing veterinarians. The survey completed by veterinary clinics found that the primary topics that veterinarians and hospital staff requested information on were (1) telemedicine (2) sustainable business techniques for providing low-cost veterinary services and (3) how to play an active role in companion animal care and public health for vulnerable populations. With additional information on these topics, veterinarians can be more equipped to provide services to clients experiencing barriers to care for their pets.

#### Telemedicine

The pandemic highlighted the value of telemedicine in veterinary practice. Telemedicine was a critical tool for helping clients to access their veterinarian and veterinary advice from the safety of their own home. Our pet owner survey indicated that most clients who used telemedicine were satisfied with the care and interested in using this technology moving forward, though respondents who had never used telemedicine were more hesitant about using it in the future compared to those who had used it. The continued use of telemedicine could help to overcome the geographical and transportation barriers experienced by clients in remote areas, and those without transportation to travel to a clinic. A recent survey of veterinarians found that the biggest rate of telemedicine adoption in clinics during the pandemic occurred in regions of higher poverty levels, and only 20% of veterinarians reported having complications while utilizing telemedicine to perform exam visits (24). Continued use of teletriage as a resource could lessen the impact of these barriers and connect clients and patients to advice and care. However, issues with technology access and use were highlighted by the aforementioned survey as a challenge in implementing telemedicine use (24), a challenge which is likely to be greatest felt by vulnerable populations. These findings show that telemedicine should have a future in veterinary medicine and to help it become a safe and reliable method of care, education for both veterinarians and clients is essential so all parties involved can feel comfortable using these new modalities of care. Research by Widmer et al. may help motivate the veterinary community to advance these efforts, as they showed that pet owners were willing to pay over $30 more for telemedicine services with their primary veterinarian over services with an online veterinarian outside of their community (25).

Other recent research has found that clients in rural areas were highly interested in carrying out treatment for pets with chronic health conditions via telemedicine platforms, and these clients were highly motivated to utilize telemedicine due to the reduced amount of stress it would cause in their pets (26). This indicates that clients can transition to the new technology with support and confidence from their veterinary team. The veterinary team can develop this confidence through increased education on the legality and efficient uses of telemedicine leading to better and more frequent communication with clients and a subsequent increase in access to care. In fact, most respondents in the veterinary survey thought that telemedicine instruction should be added to the veterinary curriculum.

Veterinary concerns were raised in our study regarding the standard of care and misdiagnosis with the use of telemedicine, a finding consistent with other surveys (27, 28). This persistence in uncertainty and lack of knowledge on the use of telemedicine and its legalities is not in-line with the increase in use in veterinary medicine, and therefore there is a need for the veterinary profession to actively promote and encourage educational opportunities in both veterinary school curriculums as well as continuing education for veterinarians to feel both comfortable and empowered to use telemedicine appropriately and effectively in their practice. Additionally, the legalities of telemedicine use in veterinary medicine could be more clearly communicated to the veterinary community by the veterinary state boards and policy makers. Finally, strategic use of the “telemedicine platform,” specifically for teleconsultations, teletriage and E-prescriptions, could help to assuage the primary concerns noted by clients and veterinarians regarding standard of care, misdiagnosis, and the inability to perform a physical examination.

#### Support of Clients Experiencing Financial Strain

Helping clinics to establish sustainable financial systems will not only allow for increased outreach to vulnerable groups during normal times but will also help them be better equipped to handle moments in time when increased client vulnerability results in a higher strain, such as during this pandemic. The third most reported reason for not seeking veterinary care was financial cost, and although this cannot be directly tied to the pandemic, a greater percent of vulnerable pet owners reported this issue than non-vulnerable pet owners. Although financial constraints were not the top reason for not seeking veterinary care, the cost of caring for their pet overall was the most frequently reported reason for considering surrender of their animal during the pandemic. A recent survey of pet owners additionally found that pet owners had concerns over the ability to afford veterinary care now (41.9%) and in the future (45%) (29). Another recent (2021) study more specifically interviewed low-income pet owners and found cost to be a significant barrier to accessing veterinary care during the pandemic (23). On the veterinary professional side, almost half of respondents indicated that their practice received increased requests for reduced cost services during the pandemic and the majority were unable to fulfill these requests. Furthermore, during this time of marked increase in financial strain, access to care organizations providing low- to no-cost veterinary services reported a marked reduction, or complete cessation, in available services during the study period. This gap in coverage could be addressed through teaching veterinarians to provide financially accessibly services through continuing education or veterinary curriculum courses. An example curriculum is The Ohio State University Preparing for Excellence in General Veterinary Medicine Program, which is designed to provide students with opportunities and build confidence in providing a “spectrum of care” in order to be prepared with these requests and financial realities upon graduation (30). Additionally, veterinary students at Lincoln Memorial University's College of Veterinary Medicine demonstrated more confidence in offering incremental care treatment options to their clients after participating in an online module focused heavily on access to care (31). Access to courses that describe the development of incremental care business models or methods to provide low costs services to clients would help clinics provide care to all pet owners in a manner that is more financially attainable. Community-medicine clinics have already been shown to increase access to veterinary care, as suggested by a recent survey study revealing that around half of pet owners visiting community-based veterinary medicine clinics had never received veterinary care for their pet before (32).

#### Understanding Issues Facing Vulnerable Populations

Education on health disparities and access to care issues faced by certain communities would help veterinarians provide service in this area. There is an opportunity here for integration and collaboration with access to veterinary care groups, who would have valuable insight into providing low-cost veterinary care. As one interviewee noted, “Many of the challenges that our communities are facing during the pandemic are basically exacerbations of challenges that already existed." Education is needed to work with vulnerable populations and those experiencing access to care issues. When interviewees from access to care organizations were asked what they learned during the pandemic about vulnerable populations and/or the support they need, there was a resounding sentiment of a new appreciation of the extent of the barriers, disparities, and lack of resources that these populations face. This sentiment was echoed by the veterinary professionals surveyed, as over half said that the pandemic changed the way that they viewed health and economic disparities in vulnerable populations. A developed curriculum for these topics could help veterinarians connect these communities with animal health resources. Critical curricular themes in addition to the inclusive business models mentioned above include public health and veterinary care for vulnerable populations, strategies for inclusive engagement with vulnerable populations, improving in-clinic perceptions of vulnerable populations, ways to crowd-source organizational aid for veterinarians, and ways to improve community partnerships with clinics to help aid vulnerable populations. Additionally, other researchers have proposed incorporation of a trauma-informed model (33) designed to combat unconscious bias while working with low-income populations (23). While organizations and groups are focusing on access to care, incremental care and support of owners experiencing these challenges, information is not equally available to general practitioners in veterinary practice, and an effort to share knowledge and approaches to these methods of client support is necessary to help bolster the traditionally and newly vulnerable pet owners as the impacts of the pandemic continue. As illustrated by Fingland et al. (30), research and support exist for spectrum of care to improve access to care across financial barriers. The next steps are to help our practicing profession build confidence and feel supported in carrying out this approach to care in a successful clinical model. Integrating this research and methodology into veterinary curricula and CE events are important steps to achieving these goals.

### Community of Care

Better networking and connections would improve communication, adaptation and support across organizations, regions, and clinics. Less than a quarter of interviewees from access to care organizations cited the use of information from the AVMA or animal shelter organizations as a resource to guide decision making during the pandemic. Further, when these interviewees were asked about support, they could have used during the pandemic, they expressed the need for resources on best practices for access to care groups during the pandemic, or information on what similar groups were doing. This highlights a need for increased collaboration and continuity within the access to veterinary care community, as well as a need for better dissemination of resources that currently exist. The networking and dissemination should be accessible beyond the veterinary profession, to include technicians and other volunteers and Access to Veterinary Care program coordinators that to not have a DVM degree, as these groups contain interested participants from varied professional backgrounds. Though distinct vulnerable populations experience different barriers and differ from each other in many ways, our research shows that access to care groups, regardless of what type of population they support, faced similar experiences during the pandemic (i.e., mental health challenges and logistical planning obstacles). There needs to exist a better infrastructure for these groups to connect with each other, allowing for efficient and valuable communication. The benefits of such a support network expand beyond the pandemic, as it could expand the network of access to care and allow for idea exchange and innovation. If such a system exists, the research here shows that it is not well-known or well-utilized, as interviewees expressed the need for information on best practices or how other access to care groups were handling the pandemic. There is therefore room for creation or expansion and improvement of a robust communication infrastructure for these organizations. Since the pandemic has started there has been an increased effort to address this issue. The American Society for the Prevention of Cruelty of Animals and the University of Minnesota brought together veterinary professionals and organizations focused on this important theme at the “Engaging the Future: Access to Veterinary Care Roundtable” hosted virtually in December 2021. Additionally, Human Animal Support Services (HASS) has been hosting twice weekly virtual meetings to bring together professionals involved in access to care organizations for support and idea exchange (34). Prior to the pandemic, the creation of the Access to Care Coalition in 2016 and the subsequent Access to Veterinary Care report released in 2018 (20) was of invaluable benefit to the field and indicative of a movement to increase discussion and education on the topic. The increased spotlight that the pandemic has shed on vulnerable populations can serve as a catalyst that builds upon this effort to increase continuity and communication within the field of access to veterinary care.

In addition to the facilitation of collaboration among professionals already in the access to care field, this could also help to increase participation of other veterinary professionals who do not yet partake in access to care work but are interested. The responses from the veterinary survey showed that the pandemic has changed perceptions on vulnerability and the role that veterinary professionals would like to have in animal care for vulnerable populations. Therefore, there could be increased interest in contributing to access to care work. Organizational infrastructure and increased communication among organizations and professionals would help facilitate the participation of more veterinary professionals and help increase involvement of veterinary clinics in access to care work.

Lastly, improved communication between veterinary hospitals and access to care organizations could help to facilitate referrals for treatable disease processes or injuries that otherwise would result in euthanasia due to cost of treatment. One study has shown that 97% of surveyed high quality, high volume spay-neuter clinics were willing to accept referrals for pyometra (35). This is a critical example of communication between organizations about the availability of low-cost services in the face of an emergency could help owners seek affordable care and avoid making a cost-based euthanasia decision.

### Future Preparedness

Looking at the future, it is critical that the veterinary and animal health professions integrate not only the educational and collaborative pieces mentioned previously but also remain aware of pre-existing issues that have been exacerbated by the pandemic. With cessation of critical services by access to care organizations, interviewees worried about unchecked animal population growth and the spread of infectious disease in areas where services focused on spay/neuter and preventive medicine services were halted. There was also concern about the public health, well-being, or mental health of community members, a notable concern considering the discussion of the disproportionate burden vulnerable populations are bearing in the face of COVID-19. Veterinarians play a crucial role in public health and the prevention of zoonoses, and in a time when disease transmission from animals has become a center of discussion among top public health officials, the veterinarian's role is now more important than ever. The pandemic has also brought to the forefront of discussion the vast disparities and inequalities in access to resources that result in vulnerable populations being disproportionately incapable of coping with and adapting to hardships (8–10). Studies have demonstrated that the prevalence of zoonotic disease is higher in underserved areas such as low-income urban areas, disadvantaged, rural populations, and areas of low socioeconomic status (36–38). The issue of zoonoses and inequalities in resources converge with each other in the very places where the vulnerable populations supported by the groups represented in these interviews exist. The pandemic could serve to spur a greater focus on zoonoses and underserved populations, while needing to address that the halt in veterinary services could cause an increase in unwanted populations and disease spread. Future responses by governments to permit these essential animal health activities will support population maintenance and disease control and lead to improved health in both human and animal populations.

Given the extensively documented and widely acknowledged issue of mental health and high suicide rates in the veterinary profession (39–41), the topic of the mental health impact of the pandemic on those who work in access to care organizations warrants proper discussion. Interviewees from access to care organizations noted that they could have used mental health support for their staff because of the toll that the pandemic was taking on the organization, and many worried about the possibility of COVID-19 transmission to the communities they served when services resume. These findings bring our attention for mental health support for the veterinarians associated with these groups to all those who work in this segment of animal care, including technicians, volunteers and all team members involved. For people who have dedicated their career to helping to support vulnerable populations, it is not surprising that the idea that delivering their support services could ultimately harm the community (as a result of transmission of disease) is a heavy weight to bear. Almost half of the interviewees said that not being able to provide normal services to the communities was the hardest part of the pandemic, and 50% of them went on to elaborate upon the mental health toll of knowing that there was animal and community suffering because they were not there. Interviewees also discussed the strain of keeping up with the high demand for services or not being able to help everyone in need. As one interviewee observed, “I think that it's important to take into account what toll this is taking on our profession … I think this is likely to have a long-term impact on our already troubled suicide rate.” In preparation for future global health disruptions and responses, it will be critical to develop and implement methods and approaches to providing support not only to the clients and communities impacted but to the health care professionals providing services. This could be accomplished through networks, collaboration, education on mental health and self-care, and by organizations creating systems of response and self-care for future instances.

### Limitations

Surveys and interviews for this project were all conducted in the first few months after the start of the COVID-19 pandemic, and associated restrictions, began in the United States. To better understand the issues that arose or persisted later in the pandemic, follow up surveys and interviews with participants would be helpful. Amazon's MTurk was used to disseminate the survey to pet owners used in this study. MTurk respondents have been shown to be more representative of the US population than those found using convenience-sampling (42). Studies have shown that respondents on MTurk tend to be younger and live in more urban areas, which is a potential limitation to representing the general population of pet owners, this also limits the potential for representation of the new and traditionally vulnerable population we sought to understand, such as older individuals and those without internet access (43). Of the respondents to the pet owner survey 27.1% reported being at high risk to COVID-19 and therefore fit into the newly vulnerable category. When added to those vulnerable based on demographic characteristics, a total of 71% of the survey respondents were categorized as vulnerable. Nonetheless, it is critical to be aware that the voices of certain vulnerable populations were not captured in the results of this study, specifically pet owners without access to the internet (for example, unsheltered and geographically isolated pet owners). To address sampling and response bias from the pet owner survey, we conducted interviews of access to care organizations who work with a range of pet owners and communities in an attempt to understand the challenges experienced by the vulnerable populations they serve. Findings were broadly consistent with survey data, however future studies should work to distribute and collect surveys, both electronically and physically, with the clientele of access to care organizations. The researchers acknowledge the limited number of access to care organizations represented. Future studies could involve interviewing additional organizations to broaden the scope of experiences shared to those from an expanded list of geographical regions, barriers encountered and professional viewpoints (beyond veterinary).

Given the opportunistic dissemination of the veterinary survey and interviews with care providers, the potential for response bias exists. Similarly, even though the veterinary clinic survey was anonymous, it is possible that participant responses were influenced by what they perceived to be a socially desirable manner. Finally, this preliminary work focuses largely on perceptions which may not accurately reflect an individual's willingness to operationalize these activities. Additional research into access to veterinary care should include exploring perceptions in animal care professionals who may not self-select to participate in this work and more participatory exercises focused on implementation. A critical step to fill these gaps in knowledge and information availability would be for access to care organizations to build upon the progress made by the 2018 Access to Veterinary Care report (20) and gather to continue to discuss relevant and emerging topics, determine common terminology and themes and develop materials and information to share.

### Summary

While the COVID pandemic has, and continues to, put significant strain on the ability of the veterinary profession to provide access to animal care, it has also helped to identify areas of improvements and ways to prepare for future crises. Education of veterinary students and professionals on the implementation and legality of telemedicine, business models to support financial resilience and client support, and understanding of improved approaches to incremental and community veterinary medicine can help improve access to care for communities experiencing barriers. Networks, collaboration and communication between veterinarians and access to care organizations can also improve responses and narrow the gaps in access to care. Finally, as a profession, it is important to advocate for the essential nature of preventive medicine should services be halted in future responses by governmental agencies, and to be aware and prepared for the dramatic impact on mental health and act to support the individuals and teams of professionals in enduring the prolonged response.

## Data Availability Statement

The original contributions presented in the study are included in the article/Supplementary Material, further inquiries can be directed to the corresponding author/s.

## Ethics Statement

The studies involving human participants were reviewed and approved by Research Integrity and Compliance Review Office, Institutional Review Board of Colorado State University. The participants provided their written or verbal informed consent for all studies.

## Author Contributions

DF and CD obtained grant funding for the research project and oversaw the research team. SS and ZG participated in the entire research process including study design, execution, data analysis, and preparation of initial drafts. DF and CD contributed to final drafts and DF finalized and submitted the manuscript. All authors contributed to the article and approved the submitted version.

## Funding

Funding for this research was provided through a grant from PetSmart Charities.

## Conflict of Interest

The authors declare that the research was conducted in the absence of any commercial or financial relationships that could be construed as a potential conflict of interest.

## Publisher's Note

All claims expressed in this article are solely those of the authors and do not necessarily represent those of their affiliated organizations, or those of the publisher, the editors and the reviewers. Any product that may be evaluated in this article, or claim that may be made by its manufacturer, is not guaranteed or endorsed by the publisher.
