# Supplementary material for: Opportunities for Expanding Access to Veterinary Care: Lessons From COVID-19
Source: Front Vet Sci. 2022 Apr 11;9:804794. doi: 10.3389/fvets.2022.804794 (PMC9036088; doi:10.3389/fvets.2022.804794)
Supplement: Supplementary file 1 [file Data_Sheet_1.DOCX]

Supplementary Material 1: Pet Owner Survey

Impact of the Covid-19 pandemic on companion animal care for pet owners

This research is being conducted by a group of veterinary students from the College of Veterinary Medicine and Biomedical Sciences at Colorado State University. The goal of this research is to investigate how the COVID-19 pandemic impacted the ability of pet owners to care for their pets and to receive veterinary care. If you are at least 18 years old have owned a dog and/or cat at some point during the COVID-19 pandemic and this pet needed veterinary care and in the 3 years prior to the pandemic, you have taken this pet to the veterinarian you are invited to complete a short anonymous online survey for which you will be paid $1. Only one respondent per IP address and MTurk number will be compensated. Your participation in this research is voluntary and you can exit the survey at any time. There are no direct benefits or known risks associated with participation in this survey. We expect the survey will take 4-8 minutes to complete. We will not collect your name or personal identifiers, and when we share the data with others, we will combine the data from all participants. Please be aware that any work performed on Amazon MTurk can potentially be linked to information about you on your Amazon public profile page, depending on the settings you have for your Amazon profile. We will not be accessing any personally identifying information about you that you may have put on your Amazon public profile page. We will store your MTurk worker ID separately from the other information you provide us. If you have any questions about the research, please contact Dr. Colleen Duncan at: (colleen.duncan@colostate.edu) or Dr. Danielle Frey at (Danielle.frey@colostate.edu) If you have any questions about your rights as a volunteer in this research, contact the CSU IRB at:  RICRO_IRB@mail.colostate.edu; 970-491-1553.

Thank you for your help!

Understanding that your responses are being collected anonymously, do you give consent to take part in this survey?

- Yes, I will take part in this survey
- No, I do not consent

Skip To: End of Survey If C1 = No, I do not consent

Q1 This survey is looking to gather information from people who fit into ALL of the following categories:

- You are 18 years or older
- At any point during the COVID-19 pandemic, you owned a dog or cat for which you were the primary caretaker (this does not include fostering a dog or cat)
- At some point during the COVID-19 pandemic, your dog or cat needed veterinary care
- In the past 3 years prior to the COVID-19 pandemic, you had taken your dog or cat to the veterinarian

Do ALL of these categories apply to you?

- Yes
- No

Skip To: End of Survey If Q1 = No

This first series of questions will help us get to know you a little bit.

Q2 What is your MTurk ID number?_______________________________________________

Q3 What state do you live in?

▼ Alabama ... Wyoming

Q4 Please specify your ethnicity

- White
- Hispanic or Latino
- Black or African American
- Native American or Alaska Native
- Asian or Pacific Islander
- Other
- Prefer not to say

Q5 The CDC defined higher risk populations for severe illness from COVID-19 as people that fall into one or more of the following categories:

- 65 years and older
- Living in a nursing home or long-term care facility
- Chronic lung disease or moderate to severe asthma
- Serious heart conditions
- Immunocompromised
- Severely obese
- Diabetes
- Chronic kidney disease undergoing dialysis
- Liver disease

Do you fit this definition of being at high risk for severe COVID-19 illness?

- Yes
- No
- Prefer not to say

We recognize that veterinary care can be expensive. This next series of questions will help us understand your financial situation so that we can better understand the cost of veterinary care for your pet.

Q6 How many people live in your household? Please include yourself in this count.

- 1
- 2
- 3
- 4
- 5
- 6
- 7
- 8
- Over 8

Display This Question:

If Q6 = 1

Q7 Which of the following best represents your annual household income?

- Below $17,500
- $17,500 - $32,000
- Above $32,000

Display This Question:

If Q6 = 2

Q7 Which of the following best represents your annual household income?

- Below $24,000
- $24,000 - $43,000
- Above $43,000

Display This Question:

If Q6 = 3

Q7 Which of the following best represents your annual household income?

- Below $30,000
- $30,000 - $54,500
- Above $54,500

Display This Question:

If Q6 = 4

Q7 Which of the following best represents your annual household income?

- Below $36,000
- $36,000 - $65,500
- Above $65,500

Display This Question:

If Q6 = 5

Q7 Which of the following best represents your annual household income?

- Below $42,500
- $42,500 - $76,500
- Above $76,500

Display This Question:

If Q6 = 6

Q7 Which of the following best represents your annual household income?

- Below $48,500
- $48,500 - $88,000
- Above $88,000

Display This Question:

If Q6 = 7

Q7 Which of the following best represents your annual household income?

- Below $54,500
- $54,500 - $99,000
- Above $99,000

Display This Question:

If Q6 = 8

Q7 Which of the following best represents your annual household income?

- Below $61,000
- $61,000 - $110,500
- Above $110,500

Display This Question:

If Q6 = Over 8

Q7 Which of the following best represents your annual household income?

- At or below $110,500
- Above $110,500

Q8 During the COVID-19 pandemic, did you lose your employment?

- Yes
- No
- Prefer not to say

Q9 During the COVID-19 pandemic, have you had health insurance?

- Yes, during the entire pandemic
- Yes, during part of the pandemic
- No
- Prefer not to say

This next series of questions will help us understand how the pandemic affected your ability to receive veterinary care for you pet.

Q10 Please select the type of veterinary care that your dog or cat needed during the COVID-19 pandemic? (select all that apply)

- My pet needed routine wellness care (checkups, vaccines, routine bloodwork)
- My pet was sick and/or injured and needed emergency medical care
- My pet needed an elective surgery (spay/neuter, dental procedure, tumor removal, etc)
- Other (please specify) ________________________________________________

Q11 During the COVID-19 pandemic, when your pet needed veterinary care, did you receive services from your veterinarian (in person or on the phone/computer)?

- Yes
- No
- Prefer not to say

Display This Question:

If Q11 = No

Q12 Please select the reasons for which you did not seek veterinary care for your pet during the COVID-19 pandemic. Select all that apply

- Financial cost
- Inability to get to the veterinary clinic
- My veterinary clinic was only offering emergency services
- Fear of getting coronavirus from staff members at the veterinary clinic
- Myself or a family member became ill and I couldn't bring my pet to the veterinarian
- Too busy, not enough time
- Prefer not to say
- Other (please specify) ________________________________________________

Q13 Please select your level of agreement with the following statements:

Taking my pet to the vet became more challenging for me during the pandemic because:

|  | Strongly agree | Agree | Neither agree nor disagree | Disagree | Strongly disagree |
| --- | --- | --- | --- | --- | --- |
| I was concerned about being able to afford the needed care for my pet |  |  |  |  |  |
| I was not able to use public transportation |  |  |  |  |  |
| I did not have access to a car |  |  |  |  |  |
| I felt increased concern about my personal health risk |  |  |  |  |  |
| I felt increased concern about the health risk to others |  |  |  |  |  |
| I felt concerned about how others would perceive me if I left my home during restrictions |  |  |  |  |  |

Q14 During the COVID-19 pandemic, did you think about surrendering your pet to a shelter, rescue, veterinary clinic, or another person/family?

- Yes, I thought about it but did not surrender my pet
- Yes, I thought about it and did surrender my pet
- No, I did not think about it
- Prefer not to say

Display This Question:

If Q14 = Yes, I thought about it but did not surrender my pet

Or Q14 = Yes, I thought about it and did surrender my pet

Q15 For what reasons did you consider surrendering your pet to a shelter, rescue, veterinary clinic, or another person/family? Select all that apply:

- Cost of caring for my pet
- Inability to obtain veterinary care for my pet
- Concerns and/or confusion over COVID-19 transmission possibilities from my pet
- Not enough time to care for my pet
- I became ill and was unable to care for my pet
- Housing-related issues
- Allergies/shedding
- Behavioral issues
- Other (please specify) ________________________________________________

Q16 Please select your level of agreement with the following statements:

|  | Strongly Agree | Agree | Neither agree nor disagree | Disagree | Strongly disagree |
| --- | --- | --- | --- | --- | --- |
| My pet has cheered me up during the pandemic |  |  |  |  |  |
| My pet has given me purpose during the pandemic |  |  |  |  |  |
| My pet has kept me active during the pandemic |  |  |  |  |  |
| During the pandemic, I never worried about getting coronavirus from my pet |  |  |  |  |  |
| I trust my veterinarian to give me information on getting diseases from my pet |  |  |  |  |  |
| I was comfortable with the idea of going to the veterinarian during the pandemic |  |  |  |  |  |

This last series of questions will help us learn if remote delivery of veterinary care would help your dog or cat receive the care it needs. This is called “telemedicine.” Telemedicine is the practice of medicine using technology to deliver care at a distance. A veterinarian in one location uses technology (such as a phone or a computer) to deliver care to a patient at a distant site.

Q17 Have you used telemedicine to receive veterinary care for your dog or cat prior to and/or during the COVID-19 pandemic?

- Yes, I used telemedicine for my pet prior to the pandemic
- Yes, I used telemedicine for my pet during the pandemic
- Yes, I used telemedicine for my pet prior to AND during the pandemic
- No, I have never used telemedicine for my pet
- I don't know if I have used telemedicine for my pet
- Prefer not to say

Skip To: Q20 If Q17 = Yes, I used telemedicine for my pet prior to the pandemic

Skip To: Q21 If Q17 = No, I have never used telemedicine for my pet

Skip To: Q22 If Q17 = I don't know if I have used telemedicine for my pet

Skip To: Q22 If Q17 = Prefer not to say

Q18 For what reasons did you utilize telemedicine to receive veterinary care during the COVID-19 pandemic? Select all that apply.

- I was worried about my risk of getting coronavirus by going to the vet
- My veterinarian recommended it
- It was easier than going into the veterinary clinic
- It is what I did before the pandemic
- Telemedicine was the only option because my vet was only open for emergencies
- Other (please specify) ________________________________________________

Q19 Would you have brought your dog or cat into the veterinary clinic to receive care during the pandemic if your veterinarian had not offered telemedicine as an option?

- Yes
- No
- I don't know
- Prefer not to say

Q20 Please select your level of agreement with the following statements:

|  | Strongly agree | Agree | Neither agree nor disagree | Disagree | Strongly disagree |
| --- | --- | --- | --- | --- | --- |
| I was satisfied with the care my pet received remotely through telemedicine |  |  |  |  |  |
| I would be interested in using telemedicine in the future (including after the pandemic is over) |  |  |  |  |  |

Display This Question:

If Q17 = No, I have never used telemedicine for my pet

Q21 For what reasons have you not used telemedicine to receive veterinary care for your cat or dog? Select all that apply

- I don’t have the technology to access it
- The technology seems too difficult to use
- My veterinarian didn’t offer it or make me aware of it
- Concerns about quality of care versus an in-person appointment
- The care my pet has needed couldn't be done with telemedicine
- Other (please specify) _______________________________________________

Display This Question:

If Q17 = No, I have never used telemedicine for my pet

Or Q17 = I don't know if I have used telemedicine for my pet

Or Q17 = Prefer not to say

Q22 Would you be interested in using telemedicine to receive veterinary care for your cat or dog in the future?

- Yes
- No
- I don't know

Thank you for completing our survey! This is the code you can enter into MTurk to receive compensation: CKPPD8341M.
