# Supplementary material for: Opportunities for Expanding Access to Veterinary Care: Lessons From COVID-19
Source: Front Vet Sci. 2022 Apr 11;9:804794. doi: 10.3389/fvets.2022.804794 (PMC9036088; doi:10.3389/fvets.2022.804794)
Supplement: Supplementary file 2 [file Data_Sheet_2.DOCX]

Supplementary Material 2: Veterinary Survey

Vulnerable Populations and Telemedicine within Veterinary Medicine

This research is being conducted by a group of veterinary students from the College of Veterinary Medicine and Biomedical Sciences at Colorado State University. The goal of this survey is to investigate the response of veterinary clinics to the needs of underserved communities and clients at high risk for severe illness from COVID-19, as well as to evaluate the use of telemedicine to increase access to care for these clients. You are invited to complete a short anonymous online survey. Your participation in this research is voluntary and you can exit the survey at any time. We will not collect your name or personal identifiers, and when we share the data with others we will combine the data from all participants. There are no direct benefits or known risks associated with participation in this survey.
We anticipate the survey will take approximately 10 minutes to complete. At the end of the survey, you will have the opportunity to enter your email for a chance to win a $50 Amazon gift card. Your email will not be linked to your survey responses, which will remain anonymous. If you have any questions about the research, please contact Dr. Colleen Duncan at (colleen.duncan@colostate.edu). If you have any questions about your rights as a volunteer in this research, contact the CSU IRB at: RICRO_IRB@mail.colostate.edu; 970-491-1553.
Thank you for your help!

Understanding that all responses collected are anonymous, do you share your consent to take part in this survey?

- Yes, I will take part in this survey.
- No, I do not consent.

Skip To: End of Block If answer = Yes, I will take part in this survey.

Skip To: End of Survey If answer = No, I do not consent.

**Demographics**

Q1 What is your role in your veterinary practice?

- Owning Veterinarian
- Associate Veterinarian
- Office Manager
- Veterinary Technician
- Other: (please specify) ________________________________________________

Q2 What is the ownership status of your practice?

- Privately Owned
- Corporate Owned
- Publicly Owned
- Non-profit (spay/neuter, street medicine, etc)
- Shelter/Rescue
- I don't know
- Other: (please specify) ________________________________________________

Q3 How old are you?

- <30
- 30-39
- 40-49
- 50-59
- 60-69
- >70

Q4 What gender do you identify with?

- Male
- Female
- Not listed
- Prefer not to say

Q5 Please select the state in which your veterinary practice is located:

▼ Alabama (1) ... Wyoming (51)

Q6 How long have you been practicing veterinary medicine?

- <5 years
- 5-10 years
- 11-15 years
- 16-20 years
- >20 years
- Not applicable

Q7 Please select which best describes the area in which your practice is located:

- Urban
- Suburban
- Rural
- Other: (please specify) _______________________________________________

Q8 Please select the answer that BEST describes your veterinary practice:

- Small animal general practice
- Small animal referral practice
- Small animal emergency practice
- Shelter/rescue
- Mixed practice, small animal focused
- Mixed practice, large animal focused
- Livestock
- Equine
- Exotics
- Other: (please specify) _______________________________________________

**Access to veterinary care for traditionally vulnerable populations:**

Vulnerable human populations have traditionally been considered as groups and communities that experience barriers to economic, political, social, and environmental resources, leaving them at higher risk for health issues for themselves and their pets. These include, but are not limited to, people experiencing homelessness, the elderly, and low income communities. The following questions relate to animal care provided to these vulnerable populations.

Q9 Please indicate how the COVID-19 pandemic changed the frequency in which your practice received the following requests related to animal care for vulnerable populations, and whether or not you were able to fulfill these requests.

|  | Degree of change: | | | Did you fulfill these requests? | | | | |  |
| --- | --- | --- | --- | --- | --- | --- | --- | --- | --- |
|  | Increased | No change | Decreased | | Yes | No | Not applicable | Wanted to but unable to | |
| Requests for reduced cost services |  |  |  | |  |  |  |  | |
| Requests for hosting mobile clinics to increase access to care |  |  |  | |  |  |  |  | |
| Requests for pet food and/or supply donations to support organizations, shelters, and/or rescues |  |  |  | |  |  |  |  | |
| Requests from shelters/rescues for low cost veterinary care |  |  |  | |  |  |  |  | |
| Other: (please specify) |  |  |  | |  |  |  |  | |

Q9a Is there anything else you would like to share about your answers to the above question?

Q10 Please select all of the following resources that would best help you to increase your ability to support vulnerable populations. Resources on:

- How to approach and interact with vulnerable populations
- How my veterinary practice can play an active role in companion animal care in these populations
- How to create sustainable systems for my practice to support clients in financial need
- The veterinarian’s role in public health for vulnerable populations
- Instruction in veterinary school on companion animal care in vulnerable populations
- None
- Other: (please specify) ________________________________________________

**Access to veterinary care for clients at high risk for COVID-19:**
The CDC defined higher risk populations for severe illness from COVID-19 as people 65 years and older, people who live in a nursing home or long-term care facility, people with chronic lung disease or moderate to severe asthma, people with serious heart conditions, people who are immunocompromised, severely obese people, people with diabetes, people with chronic kidney disease undergoing dialysis, and people with liver disease. The following questions relate to clients at high risk for severe illness from COVID-19.

Q11 Please select any changes made to your veterinary practice in an effort to better support clients during the COVID-19 pandemic (select all that apply):

|  | We made these changes  for all clients | We made these changes  for high risk clients only |
| --- | --- | --- |
| Provided house calls/ambulatory services |  |  |
| Provided remote access to care (telemedicine) |  |  |
| Modified drop off and pick up procedures |  |  |
| Enforced social distancing inside the clinic |  |  |
| Used PPE (personal protective equipment) |  |  |
| Sanitized rooms and surfaces after every use |  |  |
| Informed clients about their pet’s risk for infection and transmission of COVID-19 |  |  |
| My practice did not implement any new practices |  |  |
| Other: |  |  |

Q12 Which statement best describes how your veterinary practice plans on moving forward as pandemic recovery continues, specifically regarding your clients **at high risk for severe COVID-19 illness?**

- My practice plans on continuing to implement the practices put in place
- My practice plans on phasing out the practices put in place
- My practice plans on developing new strategies to adequately address their needs
- My practice has not decided what to do
- My practice did not put practices into place and does not plan to
- I don’t know what my practice is planning on doing
- Other: (please specify) ________________________________________________

Q13 Please select the degree to which you agree or disagree with the following statements:
 **The COVID-19 pandemic has changed:**

|  | Strongly Agree | Agree | Neutral | Disagree | Strongly disagree |
| --- | --- | --- | --- | --- | --- |
| The way that I view health and economic disparities in vulnerable populations. |  |  |  |  |  |
| The way that I view companion animal care for vulnerable populations. |  |  |  |  |  |
| The way in which I evaluate client vulnerability. |  |  |  |  |  |
| The way that I view barriers to access to veterinary care. |  |  |  |  |  |
| My perceived role in supporting vulnerable clients. |  |  |  |  |  |
| My perceived role in public health. |  |  |  |  |  |
| The role I would like to have in companion animal care for vulnerable populations. |  |  |  |  |  |

Q14 Please add any other perceptions related to your profession that have changed as a result of the COVID-19 pandemic:

Q15 Is there anything else you would like to share with us about access to care for vulnerable populations or for clients at high risk for severe COVID-19 illness?

**Telemedicine**

The AVMA defines telehealth as an umbrella term to describe the use of any technology to remotely deliver health information, education, or care. Many forms of telehealth have long been used in veterinary medicine and have few legal ramifications. These include:

- - Teleconsulting: remotely communicating with a specialist
  - Telecommunicating: remotely communicating with your own team
  - Teletriage: recommending whether or not a client should come into your clinic via the phone
  - Tele-adoptions: utilizing remote services to facilitate the adoption process
  - Other forms of telehealth have legal ramifications and require a VCPR recently established through an in-person examination of the patient. These include:
  - Telemedicine: observing and diagnosing a patient’s disease remotely
  - E-Prescriptions/e-VFD: remotely prescribing medications for companion or production animals

The **following questions relate to telemedicine in veterinary medicine.**

Q16 Please select the option that best describes how the COVID-19 pandemic impacted your opinion on telemedicine:

- Significant increase in interest
- Increase in interest
- No change
- Decrease in interest
- Significant decrease in interest

Q17 Did you use telemedicine during the COVID-19 pandemic?

- Yes
- No
- I don't know

Skip To: Q21 If Q17 = No

Q18 Please select the telehealth or telemedicine services you offered prior to and/or during the COVID-19 pandemic:

|  | Prior to | During |
| --- | --- | --- |
| Teletriage |  |  |
| Teleconsultations |  |  |
| Tele-adoptions |  |  |
| Post-operative recheck exams |  |  |
| Hospice care |  |  |
| E-prescriptions |  |  |
| Urgent Care Exams |  |  |
| None |  |  |
| Other: (please specify) |  |  |

Q19 Please select all of the reasons for which your veterinary practice has implemented telemedicine:

- My practice is using telemedicine for every client
- To increase access to care for high risk clients
- To protect the health of employees
- To abide by social distancing rules
- To supplement lack of in-person appointments
- To reach remote populations
- I don't know
- Other: (please specify) ________________________________________________

Q20 As pandemic recovery continues, will your veterinary practice continue using telemedicine to provide veterinary care for clients?

- Yes, but only for clients at high risk for severe COVID-19 illness
- Yes, for all clients
- No
- I don't know

Q21 Did you learn about telemedicine in your veterinary medicine curriculum?

- Yes
- No
- I don't know

Display This Question:

If Q1 = Owning Veterinarian

Or Q1 = Associate Veterinarian

Or Q1 = Veterinary Technician

Do not display this

if Q1 = Office Manager

or Q1 = Other: (please specify)

Q22 Do you think that veterinary medical schools should make changes to curriculum content as a result of the COVID-19 pandemic?

- Yes
- No
- I don't know

Display This Question:

If Q22 = Yes

Q23 Please select the degree to which you agree or disagree with the implementation of the following additions to the veterinary curriculum:

|  | Strongly agree | Agree | Neutral | Disagree | Strongly disagree |
| --- | --- | --- | --- | --- | --- |
| Instruction on telemedicine use |  |  |  |  |  |
| Increased instruction on public health and zoonotic disease |  |  |  |  |  |
| Instruction on vulnerabilities and barriers to access to care |  |  |  |  |  |
| Other: (please specify) |  |  |  |  |  |

Q24 Is there anything else you would like to share with us about your thoughts and/or experiences with telemedicine? _______________________________________________________________
