# Supplementary material for: Opportunities for Expanding Access to Veterinary Care: Lessons From COVID-19
Source: Front Vet Sci. 2022 Apr 11;9:804794. doi: 10.3389/fvets.2022.804794 (PMC9036088; doi:10.3389/fvets.2022.804794)
Supplement: Supplementary file 3 [file Data_Sheet_3.DOCX]

Supplementary Material 3: Access to Care Organization Interview Questions

**Background**

1. Why did you choose to work with the given population you support?
2. Will you describe the barriers to veterinary care that the population you support experiences?

(if confused with the word barriers – give the following examples)

- 1. Geographical
  2. Financial
  3. Spiritual ideology
  4. Fear of being judged
  5. Emotional/health trauma
  6. Etc.

1. What services did your organization provide prior to the COVID-19 pandemic?

**During the pandemic**

1. What services, if any, have you been able to provide during the pandemic?
2. If you stopped providing services during the pandemic, why?

(provide the following examples to demonstrate meaning of question)

- 1. Mandates (governmental and public health restrictions)
  2. Concerns over giving the disease to the people you are serving
  3. Putting your team at risk
  4. Changes in funding
  5. Lack of PPE

1. How has your organization adapted operations to support communities during this unique time?
2. What resources have you been using to guide your decision making (about what services to provide, when to stop, when to start) during this time?
3. What additional support did you need during the pandemic to provide animal care to the population you serve? Were you able to get this support?
4. Do you have any concerns about what will happen in the community you serve as a result of the disruption of your services?
5. What challenges to providing animal care have arisen as a result of the pandemic?
6. What opportunities for your organization have arisen as a result of the pandemic?
7. What has been the hardest part of the pandemic for you and your organization?
8. Even in these challenging times, have there been any moments that surprised you in a positive way?

**Moving Forward**

1. What does the process look like for your organization to start resuming normal services? Where are you at in that process?
2. What concerns do you have about beginning to resume normal services?
3. What support, if any, do you need to provide normal services again?
4. Have you made any changes during the pandemic that you will continue to implement moving forward?
5. What have you learned during this pandemic about vulnerable populations and/or the support they need that you did not know or had not thought about before?
6. Is there anything that you would like to add that we have not addressed?
